# Ion-selective graphene nanomesh membrane for sustainable osmotic power generation

**DOI:** 10.1093/nsr/nwag026

**Published:** 2026-01-20

**Authors:** Zhipeng Gao, Yuyan Gao, Zehua Yu, Chao Ma, Duo Chen, Kang Liu, Huanyu Cheng, Yanbing Yang, Quan Yuan

**Affiliations:** College of Chemistry and Molecular Sciences, Key Laboratory of Biomedical Polymers of Ministry of Education, MOE Key Laboratory of Hydrodynamic Transients, School of Power and Mechanical Engineering, Institute of Molecular Medicine, Renmin Hospital of Wuhan University, Wuhan University, Wuhan 430072, China; Department of Engineering Science and Mechanics, The Pennsylvania State University, University Park, PA 16802, USA; College of Chemistry and Molecular Sciences, Key Laboratory of Biomedical Polymers of Ministry of Education, MOE Key Laboratory of Hydrodynamic Transients, School of Power and Mechanical Engineering, Institute of Molecular Medicine, Renmin Hospital of Wuhan University, Wuhan University, Wuhan 430072, China; State Key Laboratory of Chemo and Biosensing, College of Chemistry and Chemical Engineering, College of Materials Science and Engineering, Hunan University, Changsha 410082, China; College of Chemistry and Molecular Sciences, Key Laboratory of Biomedical Polymers of Ministry of Education, MOE Key Laboratory of Hydrodynamic Transients, School of Power and Mechanical Engineering, Institute of Molecular Medicine, Renmin Hospital of Wuhan University, Wuhan University, Wuhan 430072, China; College of Chemistry and Molecular Sciences, Key Laboratory of Biomedical Polymers of Ministry of Education, MOE Key Laboratory of Hydrodynamic Transients, School of Power and Mechanical Engineering, Institute of Molecular Medicine, Renmin Hospital of Wuhan University, Wuhan University, Wuhan 430072, China; Department of Engineering Science and Mechanics, The Pennsylvania State University, University Park, PA 16802, USA; College of Chemistry and Molecular Sciences, Key Laboratory of Biomedical Polymers of Ministry of Education, MOE Key Laboratory of Hydrodynamic Transients, School of Power and Mechanical Engineering, Institute of Molecular Medicine, Renmin Hospital of Wuhan University, Wuhan University, Wuhan 430072, China; College of Chemistry and Molecular Sciences, Key Laboratory of Biomedical Polymers of Ministry of Education, MOE Key Laboratory of Hydrodynamic Transients, School of Power and Mechanical Engineering, Institute of Molecular Medicine, Renmin Hospital of Wuhan University, Wuhan University, Wuhan 430072, China; State Key Laboratory of Chemo and Biosensing, College of Chemistry and Chemical Engineering, College of Materials Science and Engineering, Hunan University, Changsha 410082, China

**Keywords:** graphene nanomesh membrane, ion-selective transport, ionic rectification, osmotic power generation

## Abstract

Atomically thin 2D membranes with minimum ion transport pathways and low ion transport resistance are ideally suited for constructing ion-selective membranes for electric power generation, and have attracted considerable recent interest. However, the practical applications of such 2D membranes for electric power generation have been severely limited due to the lack of nanoporous 2D membranes with narrow distributed nanopore arrays and sufficient charge density. Here, we report a centimeter-scale ultrathin graphene nanomesh (GNM) membrane with narrow pore size distribution (∼1.5 nm) and rich in carboxylic groups (GNM–COO^−^) for efficient osmotic power generation. The high-density nanometer pores anchored by negatively charged carboxylic groups allow efficient transport of K^+^ while selectively blocking Cl^−^. We show that the GNM–COO^−^ membrane with asymmetric charge structure exhibits a diode-like ionic rectification property and facilitates directional ion transport. When employed as an ion-selective membrane for osmotic power generation, the designed GNM–COO^−^ membrane delivers an exceptionally large output power density (175.1 W m^−^^2^) at a 50-fold salinity gradient, and retains stable power generation performance for 2 months. This work provides a strategy to develop high-performance ion-selective membranes for the sustainable harnessing of blue clean energy.

## Teaser

Graphene nanomesh membranes with uniform 1.5 nm pores and rich in carboxyl groups (GNM–COO^−^) enable ultrahigh osmotic power generation (175.1 W m^−2^) via selective cation transport and ionic rectification, achieving stable performance over 2 months, and advancing blue energy harvesting.

## INTRODUCTION

Extracting the Gibbs free energy generated at the interface between salty water and fresh water with reverse electrodialysis is considered a promising technique for sustainable and clean electricity [1,2]. As the most important component in reverse electrodialysis, ion-selective membranes that can allow selective transport of cations/anions play a central role in determining the electric power output [3,4]. Achieving efficient power generation simultaneously requires high ion selectivity and high ion conductivity. Since the ion permeability scales inversely with membrane thickness, nanoporous atomically thin 2D materials with minimum ion transport pathways and low ion transport resistance are considered ideal candidates for constructing ion-selective membranes with ultrahigh ion conductivity [5–9]. An extrapolation from a single transmembrane boron nitride nanotube measurement has suggested that nanoporous atomically thin 2D materials could deliver an extremely high power density of ∼10^6^ W m^−^^2^ [10]. Experimental investigations have also demonstrated the exciting potential of single-layer 2D materials such as graphene, molybdenum disulfide and boron nitride in power generation [11], although studies to date have typically been limited to single-layer 2D materials with a single nanopore or micro-scale nanoporous materials, which is far from practical for implementation.

Since the ion conductivity of atomically thin 2D membrane scales with the number of nanopores (*G*_m_ = $\sqrt{(N \times Gs)}$), the extension of a single nanopore demonstration to optimized nanopore arrays is essential for scalable power generation [12]. A typical demonstration is the construction of an anion-selective nanoporous covalent organic framework and a few layers of nanoporous carbon membrane for osmotic power generation [13,14]. However, a nanoporous membrane would inevitably feature a finite pore size distribution and increased concentration polarization that could compromise the ion selectivity [15,16]. A potential strategy to mitigate this challenge is to introduce asymmetric charge around the nanopores to enhance the ion selectivity.

Herein, we report a scalable atomically thin graphene nanomesh (GNM) with a high-density of nanometer pores anchored by abundant carboxylic groups (–COO^−^). The GNM–COO^‒^ membrane can facilitate selective cation transport, enabling a diode-like ionic rectification property to facilitate directional ion transport. Our studies showed the GNM–COO^−^ membrane allows efficient K^+^ transport while selectively blocking Cl^−^. Molecular dynamics (MD) simulations reveal that the high cation selectivity is associated with interactions between –COO^−^ and K^+^ that lead to a low energy barrier for K^+^ transportation. As a cation-selective membrane for osmotic power generation, the GNM–COO^−^ membrane delivers a prominent high output power density (175.1 W m^−^^2^), and retains sustainable power generation for up to 2 months. Our design defines an efficient ion-selective system for ion/molecule separation, nanofluidic control, power generation and energy extraction.

## RESULTS AND DISCUSSION

### Fabrication and structural characterization of GNM–COO^‒^

**Figure 1.**
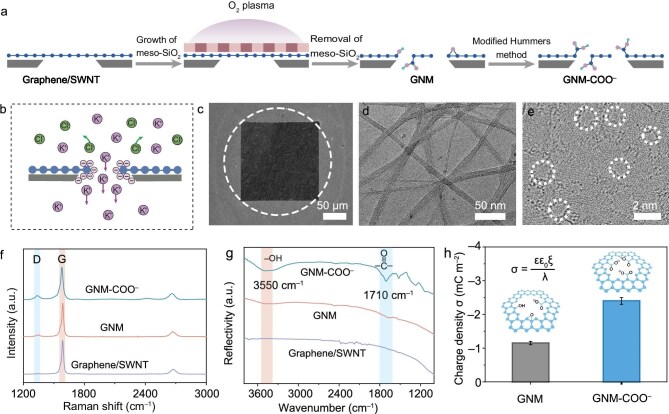
Fabrication and structural characterizations of GNM–COO^−^. (a) Schematic illustration of the fabrication processes of GNM and GNM–COO^−^ membranes. (b) Scheme of ion transport through the GNM–COO^−^ membranes under salt gradient. (c) SEM image of GNM–COO^−^ covering an Si aperture. (d) TEM image of the GNM–COO^−^ membranes. (e) Aberration-corrected TEM image of GNM–COO^−^ after 15 s of O_2_ plasma etching. The white dashed circles highlight the pores present in the GNM–COO^−^. (f) Raman spectra and (g) FTIR spectra of graphene/SWNT membrane, GNM and GNM–COO^−^ membranes. (h) Surface charge densities of GNM and GNM–COO^−^ membranes calculated from the measured membrane zeta potentials based on Gouy–Chapman theory. The error bars represent the data acquired from three individual membranes.

The GNM–COO^−^ membranes with narrow pore size distribution were fabricated through template-assisted etching followed by a surface chemistry engineering process (Fig. 1a). Briefly, chemical vapor deposition (CVD)-grown graphene was firstly transferred onto an interconnected network of single-walled carbon nanotubes (SWNTs) to form a mechanically robust membrane. Subsequently, uniform nanopores were introduced into the graphene using a meso-SiO_2_ template combined with O_2_ plasma exposure (Fig. S1) [7]. A modified Hummers method was employed to convert hydroxyl and epoxy groups at the pore edges of nanoporous graphene into carboxylic groups to produce the GNM–COO^−^ membrane with a high negative charge density that can facilitate selective cation transport (Fig. 1b) [17].

The microscopic structure of GNM–COO^−^ was comprehensively investigated with transmission electron microscopy (TEM) and scanning electron microscopy (SEM). Low-magnification TEM images show that the GNM–COO^−^ membrane was well supported by the SWNT networks with openings in the order of 10 nm (Fig. 1c and d, Fig. S2). Such compartmentalized support is essential for retaining the mechanical strength of the single-atom-thin graphene over a macroscopic scale (Fig. S3). Figure S4 shows that the freestanding centimeter-scale GNM–COO^−^ membrane retains structural integrity without obvious cracks. The Young’s modulus of the membrane measured by atomic force microscopy (AFM) is ∼5–10 GPa (Fig. S5), which is sufficient for using as an ion-selective membrane for osmotic power generation.

The aberration-corrected TEM images reveal the presence of narrow distributed nanopores in the GNM–COO^−^ membranes (Fig. 1e, Fig. S6). The average pore size and pore density were measured to be 1.5 nm (statistically analyzed from over 200 pores across multiple aberration-corrected TEM images) and ∼2.6 × 10^12^ cm^–2^ with 15 s of O_2_ plasma etching. The pore size matches well with the predicted optimal pore size (1–2 nm) for K^+^ transport while rejecting Cl^–^, making it suitable for osmotic power generation, as pores of this size are small enough to enhance charge-based selectivity via electrostatic interactions yet large enough to maintain high ion permeability—a balance essential for efficient performance [10,11,18]. More importantly, the pore size and pore density can be readily tailored by the O_2_ plasma etching time (Figs S7–S9).

Raman spectroscopy of the membrane without nanopores shows characteristic 2D (2684 cm^−1^), G (1589 cm^−1^) peaks but no apparent D peak (1330 cm^−1^) (Fig. 1f, Fig. S10), suggesting that the pristine graphene membrane exhibits a defect-free, single-layer characteristic [19]. After introducing nanopores by O_2_ plasma and chemical modification, a notable D peak emerged with *I*_D_*/I*_G_ intensity ratios of 0.5 and 0.8, respectively, indicating the formation of defects in the GNM and GNM–COO^−^ membranes [20]. Fourier-transform infrared spectroscopy (FTIR) studies reveal that GNM–COO^−^ exhibits strong absorption peaks at 3500 and 1700 cm^–1^ corresponding to the –OH and C=O stretching vibrations respectively (Fig. 1g) [21]. This is further corroborated by the X-ray photoelectron spectroscopy (XPS) analysis (Fig. S11). Together, these results demonstrate that the chemical modification successfully introduced carboxylic functional groups.

The zeta potential of the GNM–COO^−^ membrane gradually decreases with increasing pH (Fig. S12). According to a linearized Gouy–Chapman–Stern relationship that incorporates the Debye length from the Debye–Hückel theory [22], the surface charge density (*σ*) was calculated to be −1.1 mC m^–2^ for the GNM membrane and −2.4 mC m^–2^ for the GNM–COO^−^ membrane at a pH of 7 (Equation S1, Fig. 1h). Thus, the GNM–COO^−^ membrane with the negative charge characteristic is favorable for selective transport of K^+^, while blocking Cl^–^ through electrostatic interactions. The GNM–COO^−^ membrane is hydrophilic with a contact angle of 30° (Fig. S13). Such an improved hydrophilicity would increase the interface contact with water and accelerate the ion migration through the membrane [23,24].

### Charge-governed ion transport through GNM–COO^−^ membranes

**Figure 2.**
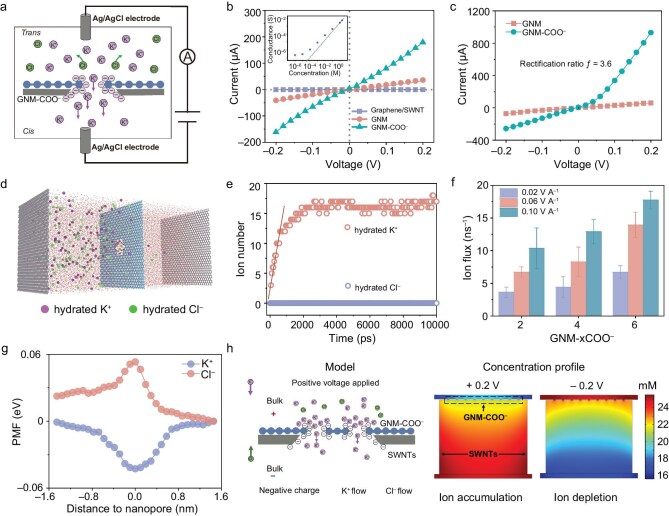
Ionic transport properties of the GNM–COO^−^ membrane. (a) Schematic of the ion transport measurement setup. Membrane was suspended over a 0.78 mm^2^ PEN aperture with the same concentration of KCl on both sides. Ions permeated through the pores of the membrane driven by an electrical potential. (b) I–V curves of the graphene/SWNT, GNM and GNM–COO^−^ membranes measured in 0.1 M KCl. Inset: the transmembrane ionic conductance of the GNM–COO^−^ membrane as a function of KCl concentration. (c) I–V curves of the GNM and GNM–COO^−^ membranes measured in 1 M KCl. (d) MD simulations on the hydrated K^+^ and Cl^–^ transport through the GNM–COO^−^ membrane. A typical simulation box used in MD simulations, showing the GNM–COO^−^ membrane (blue), hydrated K^+^ (purple), hydrated Cl^–^ (green) and water molecules in solution. (e) Numbers of hydrated K^+^ and Cl^−^ ions transferred through the GNM–COO^‒^ membrane in 0.5 M/0.01 M KCl electrolyte plotted as functions of simulation time. (f) Ion fluxes calculated for hydrated K^+^ transferred through GNM–xCOO^‒^ membrane under different electric field intensities in 0.5 M/0.01 M KCl electrolyte (where x denotes the number of carboxyl groups per pore). Error bars represent the SD with three parallel experiments. (g) PMF for hydrated K^+^ and Cl^−^ transport through the GNM–COO^−^ membrane in 0.5 M/0.01 M KCl electrolyte. (h) Numerical simulation results for the ion concentration distribution in the GNM–COO^−^ membrane, showing the accumulation (at +0.2 V) and depletion (at −0.2 V) of ions along the direction perpendicular to the membrane.

To probe the ion transport properties through the GNM–COO^−^ membrane, a free-standing membrane suspended on a polyethylene naphthalate (PEN) substrate with a 0.78 mm^2^ aperture and negligible ion-sieving capability was sandwiched between two reservoirs filled with electrolytes and a pair of Ag/AgCl electrodes (Fig. 2a, Figs S14 and S15). KCl was chosen as the electrolyte due to the similar bulk mobility and hydration radii of K^+^ and Cl^−^. Figure 2b shows the measured current–voltage (I–V) curves under a symmetric voltage of ±0.2 V when two reservoirs were filled with 0.1 M KCl solution. Before the introduction of nanopores, the graphene membrane shows almost negligible current, indicating that the membrane exhibits excellent structural integrity and ions cannot pass through the membrane. In contrast, the GNM membrane showed a linear I–V curve with a calculated conductance of 180 μS, suggesting that the presence of nanopores in the membrane allows efficient transport of ions. The GNM–COO^−^ membrane shows notably enhanced conductivity of 4.62 × 10^−5^ S m^−1^, suggesting that the presence of carboxylic functional groups at the edge of nanopores could facilitate K^+^ transport across the membranes. The GNM–COO^−^pore density calculated from I–V curves is about 1.6 × 10^12^ cm^–2^ (Equation S2), and this value is close to the pore density obtained from the aberration-corrected TEM characterizations (2.6 × 10^12^ cm^–2^). The conductance decreases non-linearly with the decrease of KCl concentration [Fig. 2b (inset), Fig. S16], indicating that the ion transport across the GNM–COO^−^ membrane is governed by a charge effect [25,26].

A diode-like I–V response was observed in the GNM–COO^−^ membrane. The rectification ratio (−0.2 V/+0.2 V) increases first and gradually decreases with a maximum rectification ratio of 3.6 upon increasing the KCl concentration from 0.1 to 3 M, further suggesting surface charge-governed ion transport (Fig. 2c, Fig. S16). In comparison, all the I–V curves of nanoporous graphene, SWNT and GNM membranes present a linear ohmic behavior with a rectification ratio (−0.2 V/+0.2 V) of about 1 (Fig. S17). The rectification phenomenon generally occurs in a nanochannel structure with heterogeneous pore size or charge distribution to improve ion conductivity [27]. Both the nanoporous graphene and SWNT membranes are symmetric in structure with relatively uniform pore size and charge distribution, and therefore these membranes show no rectification characteristics. The GNM membrane exhibits an asymmetric pore structure, while the pore size on the nanoporous graphene side (∼1.5 nm) is particularly smaller than that on the SWNT membrane side (∼30 nm). Additionally, the surface charge densities in the nanoporous graphene and SWNT sides of the GNM membrane are all relatively low, so the membranes exhibit no rectification characteristic (Figs S18 and S19). In comparison, the carboxylation modification processes introduced a large number of carboxyl functional groups at the pore edge of GNM–COO^−^ and induced the formation of a heterogeneous charge structure in the membrane, resulting in the ion rectification and ion conductivity improvement.

MD simulations were performed to understand the localized ion transport behavior across the charged GNM–COO^−^ due to the ion transport property of the membrane being mainly dependent on the nanoporous graphene layer. The simulation box is composed of hydrated K^+^, hydrated Cl^−^, water molecules and a single-pore GNM–COO^−^ with different charge densities achieved by the inclusion of 2–6 charged carboxyl groups at the pore edge (Fig. 2d). The time-dependent number of ions passing through GNM–COO^−^ under a concentration gradient of 50 (0.5 M/0.01 M KCl) shows that the number of hydrated K^+^ ions permeating through the nanopores increases from 10 to 17 ns^–1^ with increasing charge density, whereas the number of hydrated Cl^–^ ions is about 1 ns^–1^ (Fig. 2e and f, Fig. S20). This MD-predicted enhancement in K^+^ flux under a 50-fold salinity gradient is consistent with the experimentally observed increase in ionic conductance (Fig. 2b), thereby supporting the role of carboxyl groups in facilitating selective cation transport. The XY-plane average density distributions of K^+^ and O atoms of water in the charged nanopores indicate that as the charge density in the GNM–COO^−^ increases, the density distributions of K^+^ and O atoms of water gradually increase (Figs S21–S23). These results are consistent with the experimentally observed ion conductivity increase in the GNM–COO^−^ (Fig. 2b).

To gain a better insight into the K^+^ transport mechanism in the GNM–COO^−^, we calculated the potential mean force (PMF) profiles for hydrated K^+^ and Cl^−^ migration along the nanopore (*z*-axis) in the membrane (Fig. 2g, Fig. S24). The calculated energy barrier for hydrated K^+^ passing through the negatively charged GNM–COO^−^ decreases from −0.014 to −0.042 eV with increasing charge density, while the energy barrier for hydrated Cl^−^ is relatively high and continuously increases from 0.011 to 0.052 eV. The reverse trend of PMF profiles of K^+^ and Cl^–^ is due to the different electrostatic interactions between ions and charged nanopores. Negatively charged GNM–COO^−^ could attract K^+^ and reduce the energy barrier for K^+^ transport, while Cl^–^ is impeded by the charged nanopores and the energy barrier for Cl^–^ increases, thus an improved K^+^ selectivity is achieved.

We further quantified the coordination number of water molecules surrounding the hydrated K^+^ when K^+^ was passed through the GNM–COO^−^. As indicated in Figs S25 and S26, and Videos S1–S3, the coordination number of water molecules remains relatively stable during the whole transport process, suggesting that the external energy requirement originating from the dehydration effect of hydrated K^+^ is negligible. Overall, MD simulations indicate that the high charge density in the GNM–COO^−^ endow the membrane with high K^+^ conductivity and selectivity.

The ion rectification behavior of the macro-scale GNM–COO^−^ was investigated with a numerical simulation based on the Poisson and Nernst–Planck (PNP) equations. The GNM–COO^−^ with heterogeneous charge density was constructed by the inclusion of a different number of carboxyl groups in the nanoporous graphene and SWNTs. From the ion concentration profile along the direction perpendicular to the GNM–COO^−^ membrane, the presence of an ion enrichment zone under positive bias and an ion depletion zone under negative bias can be seen, and this phenomenon is consistent with the experimental results (Fig. 2h, Figs S27–S30). Under positive bias that is consistent with the chemical potential gradient direction, K^+^ and Cl^–^ preferentially transport from bulk solution into the nanopores of negatively charged GNM–COO^−^, resulting in the formation of an ion accumulation region and a higher ion conductivity. Under negative bias, K^+^ and Cl^–^ transport in the opposite direction. The ion migration from bulk solution into the nanopores is blocked due to electrostatic repulsion, thus forming an ion-depletion region. These results suggest that the heterogeneous charge structure is responsible for the rectification phenomenon in the GNM–COO^−^ (Fig. 2c). The ion rectification behavior could facilitate the ion transport and improve ion conductivity [28,29].

### Ion selectivity investigations

**Figure 3.**
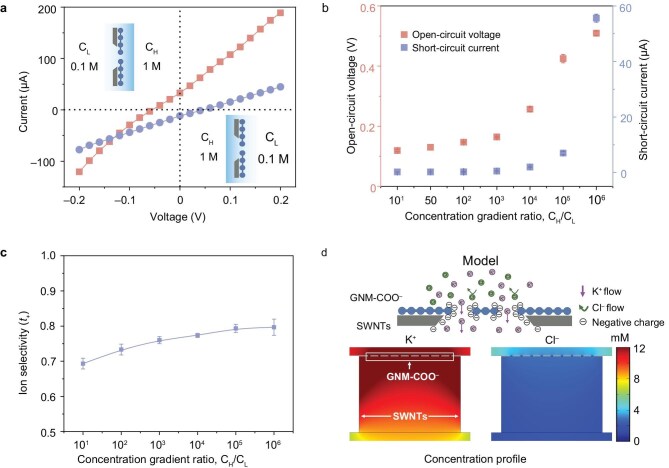
Ion selectivity of the GNM–COO^−^. (a) I–V curves of the GNM–COO^−^ at a KCl concentration gradient of 10 (1 M/0.1 M KCl). Two opposite concentration configurations were employed to explore the optimized ion transport direction. (b) The *V*_oc_ and *I*_sc_ of the GNM–COO^−^ at a series of KCl concentration gradients. (c) Calculated ion selectivity of the GNM–COO^−^ at a series of KCl concentration gradients. The error bars represent the data acquired from three individual membranes. (d) Calculated steady-state concentration distribution of K^+^ and Cl^−^ near the GNM–COO^−^ with a surface charge density of −2.4 mC m^–2^ at a KCl concentration gradient of 50 (0.5 M/0.01 M).

The ion selectivity of the GNM–COO^−^ was investigated by introducing a chemical potential gradient in the electrochemical testing system [6,28]. Since the GNM–COO^−^ is an asymmetric structure that is composed of SWNTs and nanoporous graphene, two concentration configurations were adopted to seek the optimized ion transport direction (Fig. 3a, Fig. S31). When the KCl concentrations in the nanoporous graphene and SWNT sides were 1 M (high concentration, *C*_H_) and 0.1 M (low concentration, *C*_L_), respectively, the calculated short-circuit current (*I*_SC_) from the measured I–V curve is 32.9 μA (red line). In comparison, *I*_SC_ is −11.6 μA upon the concentration gradient direction being reversed (blue line). The net current direction under two KCl-concentration configurations is consistent with the net flow of positive ions from high concentration to low concentration when the external voltage is 0 V, suggesting that the GNM–COO^−^ is selective for cations (Fig. 3a). The distinct I–V responses under different concentration configurations stem from the membrane’s asymmetric structure. The SWNT side has larger pores (∼30 nm) but a low density of negatively charged groups, introduced during acid treatment. In contrast, the nanoporous graphene side possesses much smaller pores (∼1.5 nm) but a high surface charge density. The asymmetry in both nano-structure and surface charge governs interfacial ion transport, leading to the observed direction-dependent behavior [27,29]. Since the GNM–COO^−^ exhibits relatively higher net current when the KCl concentration in the nanoporous graphene side is higher, this concentration configuration is selected for the following tests.

The energy dispersive X-ray spectroscopy (EDX) mapping indicates that the K content is particularly higher than Cl, indicating that GNM–COO^−^ exhibits excellent K^+^ selectivity (Figs S32 and S33). To further explore the ion selectivity of the GNM–COO^−^, a cyclic voltammetry (CV) test was performed to explore the transmembrane diffusion behavior of electroactive redox probes, [Ru(NH_3_)_6_]^3+^ and [Fe(CN_3_)_6_]^3^^−^. Significantly, the [Ru(NH_3_)_6_]^3+^ complex cations exhibit a considerably higher electrochemical response, with a peak current of 13.5 μA, than the [Fe(CN_3_)_6_]^3^^−^ complex anions, with a peak current of 0.4 μA (Fig. S34).

The ion selectivity performance of the GNM–COO^−^ was further evaluated by recording the I–V curves of the membrane in a series of KCl concentration gradients. As the KCl concentration gradient increases, the *I*_SC_ and open-circuit voltage (*V*_OC_) of the membranes show an increase, with maximum values of approximately 50.7 μA and 0.5 V, respectively (Fig. 3b, Table S1).

The measured *V*_OC_ consists of the diffusion potential (*E*_diff_) of ions passing through the GNM–COO^−^ and the redox potential (*E*_red_) of the Ag/AgCl electrode. To accurately evaluate the potential contribution originating from selective transport of ions, the *E*_red_ was subtracted via an electrode calibration process. Table S1 shows the *E*_diff_ and *E*_red_ of the GNM–COO^−^ at a series of KCl concentration gradients, and the maximum *E*_diff_ reaches 0.28 V. The ion selectivity of the GNM–COO^−^ can be calculated by *E*_diff_ using Equation (1):


(1) \begin{eqnarray*}
{{E_{\rm diff}\ = \ }}\left( {{\mathrm{2}}t_{\mathrm{ + }} - {\mathrm{1}}} \right)\frac{{RT}}{{zF}}{\mathrm{ln}}\left[ {\frac{{{a}_{{\mathrm{high}}}}}{{{a}_{{\mathrm{low}}}}}} \right],\end{eqnarray*}

where *F, R, T* and *z* are the Faraday constant, gas constant, temperature and ion valence, respectively. The *a*_high_ and *a*_low_ represent the ion activity at high and low KCl concentrations, respectively. The transference number *t_+_* indicates the selectivity for cations. According to Equation (1), the calculated *t*_+_ of the GNM–COO^−^ ranges from 0.69 to 0.79 depending on the KCl concentration gradient (Fig. 3c), indicating the cation-selective characteristic with *t*_+_ higher than 0.5 [29,30]. The non-linear increase of *t_+_*with the increase of concentration gradient could be attributed to the synergistic effect of transmembrane potential difference, the double electric layer and the ion rectification effect [31,32].

PNP numerical simulations indicate that the distribution of K^+^ around the nanopore is significantly higher than that of Cl^–^ in the GNM–COO^−^ membrane (Fig. 3d, Fig. S35). Additionally, compared with the GNM membrane, the GNM–COO^−^ membrane exhibits higher K^+^ concentration around the pores due to the heterogeneous charge structure, which mitigates the concentration polarization and improves the ion selectivity. The *I*_SC_ calculated from the PNP model agrees with the experimental data for the GNM–COO^−^ membrane (Fig. S36), validating the model’s predictive capability. This result reinforces the conclusion that asymmetric charge distribution and high surface charge density are key to the observed ion selectivity and rectification behavior.

### Osmotic energy conversion performance evaluation

Since the GNM–COO^−^ exhibits excellent ion selectivity and ion conductivity, the electrochemical test cell was loaded with an electrical resistor to investigate the energy conversion efficiency (Fig. S37). The output power density can be calculated by *P*_max_ *= I*^2^*R*_L_, where *I* is the current and *R*_L_ represents the load resistance. Figure S38 shows that the output power density of the GNM–COO^−^ with a testing area of 0.03 mm^2^ reaches a maximum value of 65.1 W m^−2^ at a KCl concentration gradient of 10 and an *R*_L_ of 1 kΩ (Fig. S39). The output power density could be readily tuned by the pore size of the GNM–COO^−^. Narrowing the pore size to ∼1.1 nm impedes the transport of Cl^–^, while the transport of K^+^ is also limited, thus resulting in a decreased output power density (47.3 W m^−2^). The increase of pore size facilitates the transport of both K^+^ and Cl^–^, decreasing the ion selectivity and the output power density (22.6 W m^−2^) (Fig. S40). The observed decrease in power density for smaller pores (∼1.1 nm), which are still larger than the hydrated ions, indicates that reduced pore openness increases transport resistance and potentially disrupts the functional group arrangement. This leads to a net loss in ion flux without a commensurate selectivity increase, confirming that an optimal pore size is essential for balancing selectivity and permeance. The osmotic power density of GNM–COO^−^ increases from 51.1 to 93.6 W m^−2^ when the temperature increases from 288 to 338 K (Fig. S41), following Arrhenius behavior as expected for the thermally activated ion transport in nanoconfined pores [33].

**Figure 4.**
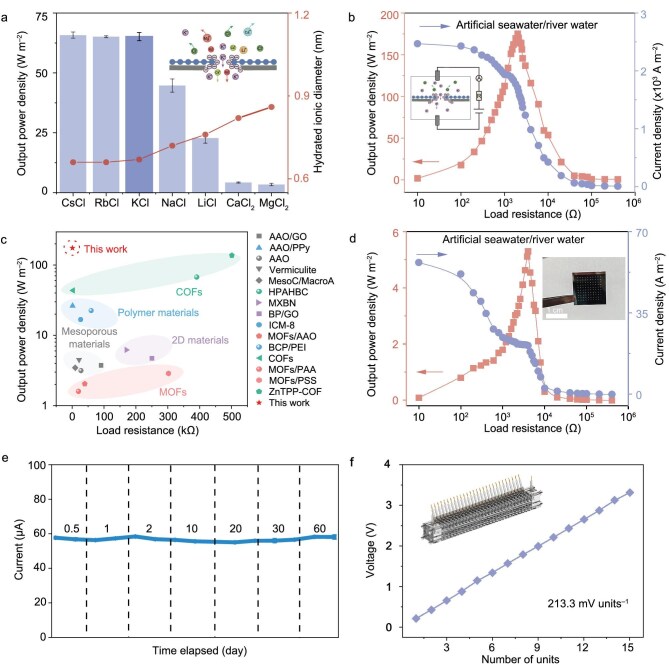
Osmotic energy generation performance of the GNM–COO^−^ membrane. (a) Output power density of the GNM–COO^−^ membrane in a variety of electrolytes, and diameter of hydrated ions of different ions. The error bars represent the data acquired from three individual membranes. (b) Output power density and current density of the GNM–COO^−^ membrane versus load resistance at a salt concentration gradient of 50 (artificial seawater/river water, 0.5 M/0.01 M NaCl). (c) Comparison of the output power density and the load resistance with those reported in the literature. (d) Output power density and current density of the GNM–COO^−^ with an effective test area of 3 mm^2^ at 0.5 M/0.01 M NaCl gradient. Inset: optical image of a representative centimeter-sized GNM–COO^−^ membrane on a porous 1.3 × 1.3 cm^2^ Si support (10 × 10 pores with 175 μm side length and 1 mm interpore distance) (e) Current–time curve of the GNM–COO^−^ membrane in artificial seawater/river water without electrolyte replenishment. The *R*_L_ is fixed at 2 kΩ. (f) The output voltage of 15 GNM–COO^−^ unit cells connected in series.

Since alkali metal ions exhibit similar sub-nanometer-sized ionic radii, the ion transport properties and osmotic energy conversion behaviors of the GNM–COO^−^ for different alkali metal ions were investigated. As shown in Fig. 4a and Fig. S42, the osmotic power density of the GNM–COO^−^ follows the order of Li^+^ < Na^+^ < K^+^ < Rb^+^ < Cs^+^ at a salt concentration gradient of 10. A maximized power density of 65.8 W m^−2^ was observed for Cs^+^, which can be ascribed to the relatively smaller hydrated ionic radii and larger cation diffusion coefficient that enables more efficient charge separation (Table S2) [13].

In the practical seawater system, there also exists an enormous quantity of Ca^2+^ and Mg^2+^ other than alkali metal ions. The osmotic energy conversion behaviors of the GNM–COO^−^ were tested in a variety of electrolytes. As shown in Fig. 4a, GNM–COO^−^ shows lower osmotic power density for divalent cations such as Ca^2+^ (4.1 W m^−2^) and Mg^2+^ (3.6 W m^−2^), which can be attributed to the large hydrated radius of divalent cations increasing the energy barrier for ion transport and decreasing the ion selectivity [13].

The osmotic power generation performance of the GNM–COO^−^ at a salt concentration gradient of 50 (artificial seawater/river water, 0.5 M/0.01 M NaCl) is determined to reach a maximum value of 175.1 W m^−2^ with an *R*_L_ of 2 kΩ (Fig. 4b). Figure 4c summarizes the osmotic power generation performance of the GNM–COO^−^ and previously reported ion-selective membranes, in terms of load resistance and output power density [13,14,29,33–43] (Table S3). The GNM–COO^−^ shows a higher output power density compared with that of most reported ion-selective membranes. The unprecedented output power density of the GNM–COO^−^ is mainly ascribed to the enhanced ion selectivity and high ion conductivity of the membrane with uniform pore size distribution and high charge density. Additionally, the ions–diode behavior of asymmetric membrane structure also effectively facilitates ion transport and further promotes the energy conversion process.

To explore the scalability of GNM–COO^−^, a macroscopic osmotic energy generator with a centimeter-scale membrane [the effective test area is 3 mm^2^, 100-fold larger than currently reported test area (0.03 mm^2^)] was constructed (Fig. S43). As shown in Fig. 4d, the GNM–COO^−^ achieves an osmotic power density of 5.3 W m^−2^. To our knowledge, this value is the highest power density reported in the literature for large-area ion-selective membranes to date and satisfies the commercialization benchmark of 5 W m^−2^ (Table S4). It is worth noting that the internal resistance of the GNM–COO^−^ remains unchanged after enlarging the test area. While the membrane’s ultrathin thickness, uniform pore size distribution and diode-like ionic rectification behavior contribute to its low inherent membrane resistance, the observed area-independent resistance suggests that the overall device resistance in the macroscopic configuration may be dominated by the access resistance arising from electrode interfaces and solution geometry, as noted in prior studies [44]. We further expanded the test area of GNM–COO^−^ to 12 mm^2^ (400 times the test area reported in the literature) and 75 mm^2^ (2500 times the test area reported in the literature). The output power density of GNM–COO^−^ was 3.3 and 1.4 W m^−2^, respectively. More importantly, the area of the GNM–COO^−^ membrane could reach up to 12 cm^2^ or even larger (Figs S44 and S45), demonstrating the macroscopic scalability of our fabrication process. These results suggest that GNM–COO^−^ is scalable both in area and osmotic power generation. The decreased power density upon enlarging the test area is probably due to the increased concentration polarization [45] and the misalignment between membrane pores and the silicon window substrate. Under the static operating conditions of our measurements, an increase in membrane area amplifies the diffusional limitation for ions traveling from the bulk solution to the membrane surface, thereby creating a thicker concentration boundary layer. This reduces the effective salinity gradient across the membrane and thus the osmotic power output. Such scale-dependent concentration polarization is commonly observed in nanofluidic and reverse electrodialysis systems, where larger membrane areas exacerbate ion transport resistance and energy conversion efficiency [1,4,11,15,46]. The engineering issues of solution storage may arise when GNM–COO^−^ membranes are applied to practical systems. Therefore, in order to scale up nanoporous membrane from micro to macro, more efforts are needed to narrow this gap, including introducing an asymmetric charge characteristic in the GNM–COO^−^ membrane to increase the ion rectification properties, as well as optimizing the structure and ion transport interface to improve ion selectivity and ion conductivity.

More importantly, the osmotic energy generator with GNM–COO^−^ maintains relatively stable *I*_SC_ and power density for 2 months without continuous electrolyte replenishment and structural destruction (Fig. 4e, Fig. S46). Compared with the commercial cellulose acetate (CA) membrane, the GNM–COO^−^ membrane shows high anti-biofouling performance to resist bacterial attachment after a long period of operation, and retains stable osmotic energy generation performance (Figs S47and S48). The efficient ion selectivity and conductivity combined with the excellent anti-biofouling characteristics promise potential of GNM–COO^−^ in the construction of industrial-scale osmotic power generator for sustainable power generation, as there is no fundamental limitation in further scaling the membrane with scalable membrane fabrication and modification technologies. The GNM–COO^−^ can be connected in series to achieve higher voltage outputs (Fig. S49). The output voltages of the GNM–COO^−^ power generator show a perfect linear relationship of 214.1 mV per unit cell, and reach up to about 3.2 V under a concentration gradient of 50 (0.5 M/0.01 M NaCl) (Fig. 4f). Notably, the GNM–COO^−^ power generator could recharge capacitors to achieve continuous electric power supply for the Global Positioning System (GPS) and obtain real-time GPS position information (Fig. S50, Video S4). These results suggest that the GNM–COO^−^ power generator has great potential for practical application as a power source and makes the osmotic power a tangible and promising alternative.

## CONCLUSION

In summary, we designed a GNM–COO^−^ membrane with dense sub-nanometer pores and abundant negative charge for efficient and selective cation transport and osmotic power generation. The GNM–COO^−^ membrane allows efficient transport of K^+^ while selectively blocking Cl^–^. The ultrathin characteristic and asymmetric charge structure of the GNM–COO^−^ promote fast and directional K^+^ transport, significantly improving ion conductivity and ion selectivity. MD simulations and PNP numerical analysis reveal that the ultra-high ion conductivity and ion selectivity of the GNM–COO^−^ membrane is mainly attributed to the significantly lower energy barrier for K^+^ to pass through the negatively charged nanopores. When used as an ion-selective membrane in an osmotic energy generator, the GNM–COO^−^ achieves high cation selectivity (0.79) and an exceptionally large output power density (175.1 W m^–2^). Moreover, the GNM–COO^−^ shows a high output power density that matches well with the requirements in practical applications after scaling the membrane to a large area. Notably, the GNM–COO^−^ retains stable power generation performance for up to 2 months. The extension of 2D membranes from demonstration of a single nanopore to investigation of nanopore arrays enables the membrane to be highly attractive for sustainable clean energy extraction.

## Supplementary Material

nwag026_Supplemental_Files
