# Supplementary material for: Ion-selective graphene nanomesh membrane for sustainable osmotic power generation
Source: Natl Sci Rev. 2026 Jan 20;13(5):nwag026. doi: 10.1093/nsr/nwag026 (PMC12976675; doi:10.1093/nsr/nwag026)
Supplement: nwag026_Supplemental_Files [file nwag026_supplemental_files.zip › Supplementary data.pdf]

Supporting Information

Ion-selective graphene nanomesh membrane for sustainable osmotic power generation

Authors:

Zhipeng Gao[1,†], Yuyan Gao[3,†], Zehua Yu[1,†], Chao Ma[2], Duo Chen[1], Kang Liu[1], Huanyu Cheng[3], Yanbing Yang[1*], Quan Yuan[1,2*]

Affiliations:

[1]College of Chemistry and Molecular Sciences, Key Laboratory of Biomedical Polymers of Ministry of Education, MOE Key Laboratory of Hydrodynamic Transients, School of Power and Mechanical Engineering, Institute of Molecular Medicine, Renmin Hospital of Wuhan University, Wuhan University, Wuhan 430072, P. R. China

[2] State Key Laboratory of Chemo and Biosensing, College of Chemistry and Chemical Engineering, College of Materials Science and Engineering, Hunan University, Changsha 410082, P. R. China

[3]Department of Engineering Science and Mechanics, The Pennsylvania State University, University Park, PA 16802, USA

[†]Equally contributed to this work.

This file includes:

Materials and Methods

Figures S1 to S50

Table S1 to S5

Video S1 to S4

References

Contents

Methods......6
Structure characterization......6
Fabrication of GNM and GNM-COO^- membranes......6
Ion transport performance investigations......7
Ion selectivity evaluations......7
Osmotic energy conversion performance of the GNM-COO^- membranes......8
Preparation and osmotic energy conversion performance of the large area GNM-COO^- membranes......8
Membrane biofouling tests......9
Calculation of surface charge density of membranes......9
Pore density of the membranes......9
Molecular dynamics simulations......10
Poisson−Nernst−Planck (PNP) simulations......10
Supplementary Figures......12
Figure S1 TEM image of the meso-SiO_2 membrane......12
Figure S2 SEM image of the GNM-COO^- membrane......12
Figure S3 AFM image and height profile of the GNM-COO^- membrane......13
Figure S4 Photograph of the freestanding centimeter-scale GNM-COO^- membrane......13
Figure S5 (A) Modulus map and (B) modulus distribution of the GNM-COO^- membrane......14
Figure S6 Aberration-corrected TEM image of graphene......14
Figure S7 (A, B) Aberration-corrected TEM image and (C) pore size distributions of GNM-COO^- membrane prepared by O_2 plasma etching time of 10 s. (A) and (B) represent two samples produced with the same conditions......15
Figure S8 (A, B) Aberration-corrected TEM image and (C) pore size distributions of GNM-COO^- membrane prepared by O_2 plasma etching time of 20 s. (A) and (B) represent two samples produced with the same conditions......15
Figure S9 (A, B) Aberration-corrected TEM image and (C) pore size distributions of GNM-COO^- membrane after Hummers' treatment prepared by O_2 plasma etching time of 20 s. (A) and (B) represent two samples produced with the same conditions......16
Figure S10 Raman spectra of graphene and SWNT membranes......16
Figure S11 XPS C1s core of (A) GNM and (B) GNM-COO^- membranes. After chemical modification, the peaks correspond to C=O functionalities for the GNM-COO^- membrane exhibit an obvious increase compared with GNM membrane......17
Figure S12 Zeta potential of the GNM-COO^- membrane as a function of pH. Error bars represent the s.d. with three parallel experiments......17
Figure S13 Contact angle of the (A) SWNT, (B) GNM and GNM-COO^- membranes......18
Figure S14 Optical images of (A) a PEN support with an aperture of 0.78 mm^{-2} and (B) a layer of GNM-COO^- covered on a PEN aperture......18
Figure S15 I-V curves of the PEN+GNM-COO^- membrane, PEN substrate, and Si substrate measured in 0.5 M KCl......19
Figure S16 (A) I-V curves of the GNM-COO^- membrane at different KCl concentrations. (B) Rectification ratio of the GNM-COO^- membrane at different KCl concentrations......19

Figure S17 I-V curves of the SWNT, nanoporous graphene and GNM membrane at 1 M KCl concentration. 20
Figure S18 (A) I-V curves of the SWNT membrane at different KCl concentrations. (B) Ionic conductance of the SWNT membrane as a function of electrolyte (KCl) concentration. 20
Figure S19 (A) I-V curves of the GNM membrane at different KCl concentrations. (B) Ionic conductance of the GNM membrane as a function of KCl concentration. 21
Figure S20 Numbers of hydrated K^+ and Cl^- ions transferred across the (A) GNM-2COO^- and (B) GNM-4COO^- membranes in 0.5 M/0.01 M KCl electrolyte plotted as functions of simulation time. 21
Figure S21 Density distribution profiles of hydrated K^+ in xy-planes for GNM-xCOO^- membrane under a concentration gradient of 50 (0.5 M/0.01 M KCl). 22
Figure S22 Density distribution profiles of O atoms of water in xy-planes for GNM-xCOO^- membrane under a concentration gradient of 50 (0.5 M/0.01 M KCl). 22
Figure S23 MD simulation of the concentration distributions of (A) K^+ and (B) Cl^- as a function of vertical distance from the pore center of GNM-xCOO^- membrane under KCl concentration gradient of 50 (0.5 M/0.01 M). 23
Figure S24 PMF profiles for (A) hydrated K^+ and (B) hydrated Cl^- transport through the GNM-xCOO^- membranes under concentration gradient of 0.5 M/0.01 M KCl electrolyte. 23
Figure S25 Coordination number of water at the selected locations when hydrated K^+ transport through the (A) GNM-2COO^-, (B) GNM-4COO^- and (C) GNM-6COO^- membranes. Error bars represent the s.d. with three parallel experiments. 24
Figure S26 Radial distribution function (RDF) of functional group for (A) GNM-2COO^-, (B) GNM-4COO^-, (C) GNM-6COO^- membranes and (D) hydrated K^+. 24
Figure S27 Schematic of the ions rectification mechanism in the GNM-COO^-. (A) Enhanced ion conductivity was observed at positive voltage; (B) low ion conductivity was observed at negative voltage. 25
Figure S28 The simulated 2D domain for PNP model. Two KCl electrolyte reservoirs are connected by a two-segment nanochannel composed of the nanoporous graphene and SWNT. In all the simulations, the concentration and potential of the central pore are used to calculate the current. 25
Figure S29 Simulated ions concentration profiles in the nanoporous graphene membrane with applied voltage bias of (A) + 0.2 V and (B) − 0.2 V. 26
Figure S30 Simulated ions concentration profiles in the SWNT membrane with applied voltage bias of (A) + 0.2 V and (B) − 0.2 V. 26
Figure S31 The (A) V_{oc} and (B) I_{sc} of the GNM-COO^- at a series of KCl concentration gradients in two concentration configurations. 27
Figure S32 EDX analysis of the GNM-COO^- membrane: (A) K and (B) Cl elemental maps, and (C) the corresponding EDX spectrum. 27
Figure S33 X-ray photoelectron spectroscopy (XPS) of the GNM–COO^- membrane: (A) full survey spectrum, (B) high resolution spectra of C1s and K2p, and (C) high resolution Cl2p spectrum. ...28
Figure S34 CV curves of the GNM-COO^-. ($[Ru(NH_3)_6]^{3+}$ serves as a cationic electroactive probe (red curve); $[Fe(CN)_6]^{3-}$ acts as an anionic electroactive probe (blue curve). 28
Figure S35 Calculated steady-state concentration distribution of (A) hydrated K^+ and (B) hydrated Cl^- near the GNM-COO^- under a salt gradient of 0.5 M/0.01 M KCl, respectively. 29
Figure S36 Comparison of the experimental I_{SC} of the GNM-COO^- with the simulated value. Error

bars represent the s.d. with three parallel experiments..29
Figure S37 Optical image of the electrochemical device. The GNM-COO$^-$ membrane on a Si support with an aperture of 0.03 mm^2 was sandwiched between two reservoirs filled with seawater/river water in each side. Ag/AgCl electrodes were employed to characterize the current-voltage response...30
Figure S38 The output power density and current density of the GNM-COO$^-$ *versus* load resistance at a salt concentration gradient of 10 (1 M/0.1 M KCl). 30
Figure S39 SEM images of (A) a Si aperture with an area of 0.03 mm^{-2} and (B) a layer of GNM-COO$^-$ covered on a Si aperture. 31
Figure S40 The output power density of the GNM-COO$^-$ prepared with different O_2 plasma etching time. Error bars represent the s.d. with three parallel experiments. 31
Figure S41 The output power density of the GNM-COO$^-$ at different temperatures. Error bars represent the s.d. with three parallel experiments. 32
Figure S42 I-V curves of the GNM-COO$^-$ at 10^{-3} M LiCl, NaCl, KCl, RbCl and CsCl electrolytes. 32
Figure S43 (A) Digital photograph of a representative centimeter-sized GNM-COO$^-$ membrane on a porous 1.3×1.3 cm^2 Si support (10×10 pores with 175 μm side length and 1 mm interpore distance). (B) Current-voltage curve of the GNM-COO$^-$ with an effective test area of 3 mm^{-2} at 0.5 M/0.01 M NaCl gradient. 33
Figure S44 Photograph of a large area GNM-COO$^-$ membrane. 33
Figure S45 The output power density and current density of the GNM-COO$^-$ membrane with an effective test area of (A) 12 mm^2 and (B) 75 mm^2 at 0.5 M/0.01 M NaCl gradient. Inset: Optical image of a representative centimeter-sized GNM-COO$^-$ membrane on a porous 1.3×1.3 cm^2 Si support..34
Figure S46 SEM images of GNM-COO$^-$ (A) before and (B) after osmotic energy stability test. ..34
Figure S47 Confocal microscopy images of *R. palustris* cells attached to the GNM-COO$^-$ membrane and CA membrane at different test time periods. 35
Figure S48 Current-time curve of the GNM-COO$^-$ membrane in artificial seawater/river (0.5 M NaCl/0.01 M NaCl, $MgCl_2$, $CaCl_2$ and *R. palustris*) water without electrolyte replenishment. The R_L is fixed at 2 kΩ...35
Figure S49 Schematic of the circuit design and working demonstration of the self-powered GPS electronic device. The circuit primarily consists of four elements: GNM-COO$^-$ power generator, capacitor, switch and GPS electronic device..36
Figure S50 (A) Schematic of a home-made self-powered positioning system; (B) A smart phone interface showing the successfully located position of the lab building in Wuhan University with the GPS. (C) A smartphone interface showing the specific location information of the lab building of College of Chemistry and Molecular Sciences in Wuhan University with the GPS. 36
Table S1. The open-circuit potential, redox potential, and diffusion potential of the GNM-COO$^-$ at different KCl concentration gradients. 37
Table S2. Ionic species and their hydrated ionic radii and bulk diffusion coefficient.....................38
Table S3. Energy conversion performance, thickness and load resistance of the GNM-COO$^-$ compared with the state-of-art membranes at a salt concentration gradient of 50. 39
Table S4. Summary of the state-of-art osmotic power density of large-area nanoporous membranes at a salt concentration gradient of 50 and other biomimetic ion channels. 40

Table S5 Atomistic coordinates for the GNM-xCOO$^-$..41
References..45

Methods

Structure characterization

The morphological structures of membranes were characterized by SEM (Zeiss Merlin Compact) and TEM (JEM-2100). Aberration-corrected TEM images were collected on a Thermo Scientific Themis Z operated at 80 kV. The Young's modulus and thickness of the GNM-COO^{-} membrane were characterized by AFM (Park nx10, Korea). Raman spectra were carried out using a Renishaw in Via. The surface element composition of the GNM and GNM-COO^{-} membranes was characterized by XPS (ESCAI AB250Xi, USA). FTIR spectra were recorded on a NICOLET 5700 FTIR spectrophotometer equipped with a Golden Gate attenuated total reflection (ATR) device. The hydrophilic/hydrophobic of the SWNTs, GNM, and GNM-COO^{-} membranes were measured using the contact angle OCA20 (KRUSS, Germany). A confocal laser-scanning microscope of the *R. palustris* cells were recorded on a Leica sp8 with a 20× objective. Energy dispersive X-ray spectroscopy of the GNM-COO^{-} membrane was measured by SEM (Zeiss Merlin Compact). Zeta potential of the GNM and GNM-COO^{-} membranes were performed on a SurPASS 3 solid Surface Zeta potential (Anton Paar, Austria).

Fabrication of GNM and GNM-COO^{-} membranes

The synthesis strategy of GNM and GNM-COO^{-} membranes were shown in fig. 1a. Initially, a single layer of CVD grown graphene on Cu substrate was used as the starting material. The graphene/SWNT membranes were obtained by transferring the SWNT membranes with porous networked structure onto the graphene surface and etching the Cu foil in a $FeCl_3$ (1 mol L^{-1}) solution for 2 h, followed by washing in water several times. For the preparation of GNM membranes, a layer of mesoporous SiO_2 (meso-SiO_2) membrane with perpendicular porous channels that serves as templates was grown by a Stober method [1]. Specifically, the graphene/SWNT membranes were immersed into a meso-SiO_2 precursor solution containing 0.08 g cetyltrimethyl ammonium bromide, 15 mL ethanol, 35 mL water, 5 µL concentrated ammonia aqueous solution and 40 µL tetraethyl orthosilicate to grow meso-SiO_2 template and reacted at 60 °C for 4 hours. The free-standing GNM membranes were fabricated by sequentially including nanopores in the GNM with O_2 Plasma etching (50 W, 20 mtorr) and removing meso-SiO_2 template with HF. The O_2 plasma treatment time was varied from 10 s to 20 s to control the pore size and pore density in the GNM membranes. The pore size distribution was determined through statistical analysis of over 200 pores from multiple aberration-corrected TEM images. The average pore size and standard deviation are reported to reflect the uniformity of the pore structure. The GNM-COO^{-} membranes with carboxyl groups were obtained by a modified Hummers method [2]. Specifically, the GNM membranes were firstly immersed into 10% KOH solution for 2 hours to activate the oxygen-containing groups at the pore edges of GNM. Next, the GNM membranes were subjected into 10% NaOH and chloroacetic, and the mixture was continuously reacted at 50 °C overnight. By sequentially washing with ethanol solution and rinsing with water to remove unreacted substances, GNM-COO^{-} membranes were obtained. Notably, the Hummers' treatment for carboxyl

functionalization did not significantly alter the pore size distribution, as confirmed by TEM analysis, ensuring that the enhanced performance stems primarily from the increased surface charge density. The SWNT possess excellent mechanical performance, freestanding characteristic, and outstanding chemical stability, enabling their capability to support the single-layer nanoporous graphene membrane to enhance the mechanical performance. These properties ensure that the GNM-COO^- membrane maintains structural stability over a large area.

Ion transport performance investigations

Electrochemical measurements were performed to investigate the transmembrane ion transport performance. Specifically, the membranes suspended on a Si substrate with a 0.78 mm^2 aperture were sandwiched between two chambers of a custom-designed electrochemical cell that contains 4.0 mL KCl solution in each cell. The KCl concentration between the two sides is consistent. The electrochemical measurement was carried out on a Keithley 6487 picoammeter/voltage source (Keithley Instruments). The I-V curves of the membranes at different KCl concentrations were recorded and the corresponding conductance were calculated to evaluate the ion transport performance of the membranes. The ionic rectification properties of the membranes was measured by sweeping voltages from – 0.2 V to + 0.2 V with a step voltage of 0.02 V, and recorded the I-V curves. The rectification ratio was calculated based on the current ratio between –0.2 V and + 0.2 V.

Ion selectivity evaluations

To investigate the ion selectivity of the GNM-COO^- membrane, a chemical potential gradient was introduced in the test system through the inclusion of different concentrations of KCl solution across the membrane. The tested membrane area is 0.78 mm^2. The GNM-COO^- membrane shows relatively low membrane resistance when the KCl concentration in the nanoporous graphene side is high. The ion selectivity was evaluated by recording the I-V curves at KCl concentration gradient ratio of 10, 50, 10^2, 10^3, 10^4, 10^5, and 10^6. The derivation of equation (1) assumes the following: (i) ideal Donnan exclusion at the pore–solution interface, (ii) negligible convective contribution to ion flux, and (iii) steady-state transport under the applied concentration gradient. This theoretical framework is widely used for characterizing ion-selective membranes in nanofluidic systems. In addition to the I-V measurements, electrochemical tests and EDX mapping were also employed to investigate the ion selectivity of membranes. To directly evaluate and visually demonstrate the cation-selectivity of the negatively charged GNM-COO^- membrane, cyclic voltammetry (CV) was performed using oppositely charged redox probes. An electrochemical workstation (CHI660E, Chenhua, Shanghai) and a three-electrode system were utilized for cyclic voltammetry measurements. The GNM-COO^- membrane was prepared on an ITO electrode to serve as the working electrode. A platinum wire and an Ag/AgCl electrode were used as the counter electrode and reference electrode, respectively. The scan rate was set at 200 mV s^{-1}. 0.1 M KCl was used as the supporting electrolyte. The anionic $[Fe(CN)_6]^{3-}$ and cationic $[Ru(NH_3)_6]^{3+}$ probes were selected to determine the ion selectivity of GNM-COO^-. The significantly higher electrochemical response (peak current) observed for

the cationic probe compared to the anionic probe provides direct evidence of preferential cation transport through the membrane, corroborating the high cation transference number obtained from diffusion potential measurements. This approach is well-established for characterizing ion selectivity in nanofluidic systems [3-5]. To qualitatively assess the cation selectivity of the GNM-COO⁻ membrane, energy-dispersive X-ray spectroscopy (EDX) mapping was performed. Prior to analysis, the GNM-COO⁻ membrane was immersed in a 0.1 M KCl aqueous solution for 12 hours to facilitate ion adsorption. The sample was then carefully retrieved and rinsed thoroughly with copious amounts of deionized water to eliminate any loosely bound or free ions from the surface. Subsequently, the membrane was transferred onto a clean silicon substrate and air-dried for SEM and EDX characterization. The relative elemental content of potassium (K) and chlorine (Cl) was analyzed from the obtained maps.

Osmotic energy conversion performance of the GNM-COO⁻ membranes

The osmotic energy conversion properties of the membranes were investigated by loading an electrical resistor in the electrochemical cell. In a typical experiment, the GNM-COO⁻ membrane was mounted between two reservoirs containing NaCl or KCl solutions with different salt concentrations. A pair of Ag/AgCl electrodes were used to sweep the applied voltage from – 0.2 V to + 0.2 V in steps of 0.02 V, and the resulting current–voltage (I–V) curves were recorded. The short-circuit current (I_{SC}) and open-circuit voltage (V_{OC}) were extracted from these curves. The output power density (P) was then calculated using the formula $P = I^2 \times R_L$, where R_L is the load resistance. The maximum output power density (P_{max}) corresponds to the value at the optimal load resistance where the product of current and voltage is maximized. All tests were performed with a consistent membrane effective area of 0.03 mm^2, in accordance with established practices in previous studies on nanofluidic osmotic energy conversion. The GNM-COO⁻ membranes with different pore sizes were measured to investigate the pore size on the osmotic energy conversion performance. The osmotic energy conversion performance of the GNM-COO⁻ membrane was also examined in various electrolytes, including LiCl, NaCl, RbCl, CsCl, $MgCl_2$ and $CaCl_2$ electrolytes at a salt concentration gradient of 10. The temperature-dependent performance of the GNM-COO⁻ membrane was tested under uniform heating of both electrolyte reservoirs, ensuring that any change in output power reflects only the intrinsic temperature dependence of ion transport in the membrane and electrolyte, without interference from thermal gradients or thermoelectric effects. The electric generation durability of the GNM-COO⁻ membrane was recorded without continuous electrolyte replenishment. The electrolyte solutions were all prepared using millipore water (18.2 MΩ cm).

Preparation and osmotic energy conversion performance of the large area GNM-COO⁻ membranes

To investigate the scalability of the GNM-COO⁻ membrane, we fabricated a centimeter-sized GNM-COO⁻ membrane with an effective test area of 3 mm^2. At this scale, the electrical double layer does not overlap, thereby rendering the silicon substrate incapable of selective ion sieving. The osmotic energy conversion

performance of the GNM-COO^- membranes were covered on a 1.3 × 1.3 cm^2 Si support with 10 × 10 holes (175 μm in side length and 1 mm between holes), namely 100 single microcells in parallel at NaCl concentration ratio of 50 (0.5 M / 0.01 M NaCl).

Membrane biofouling tests

The anti-biofouling performance of the GNM-COO^- membrane was tested with a confocal laser scanning microscope (FV1200, Olympus, Japan). *R. palustris* were cultured with (4',6-diamidino-2-phenylindole) DAPI in Luria-Bertani broth medium overnight at 37 °C. Then, the *R. palustris* were collected by centrifugation and suspended in PB buffer. GNM-COO^- membranes and CA membranes were immersed in 3 mL of bacterial solution at room temperature for different times for bacterial adhesion experiments. The GNM-COO^- membranes and CA membranes were washed with PB buffer three times before confocal fluorescence imaging.

Calculation of surface charge density of membranes

The zeta potential of the GNM-COO^- membrane was measured using the streaming potential method on a SurPASS 3 electrokinetic analyzer (Anton Paar, Austria). The membrane was mounted in an adjustable gap cell, and the zeta potential was determined from the measured streaming potential using the Helmholtz–Smoluchowski equation using the instrument's software. Measurements were conducted in 1 mM KCl solutions at varying pH values. The surface charge density (σ) [6] is estimated using a linearized form of the Gouy–Chapman–Stern relation, which incorporates the Debye length (λ_D) calculated from Debye–Hückel theory for a symmetric 1:1 electrolyte (1):

$$\sigma = \frac{\varepsilon\varepsilon_0\xi}{\lambda} \tag{1}$$

where ε is the dielectric constant of the solvent and ε_0 is the dielectric permittivity of free space, ζ is the zeta potential, and λ is Debye length. The calculation assumes a uniformly charged surface and a relatively low surface potential ($|\zeta| \lesssim 50$ mV), conditions that are consistent with our experimental measurements. This approach for determining surface charge density from zeta potential is widely used in the characterization of nanofluidic and 2D material membranes.

Pore density of the membranes

The pore density of GNM-COO^- was calculated from the conductance of the membrane. Specifically, I-V curves of PEN support and GNM-COO^- membrane supported by PEN substrate in electrolyte solution of 0.1 M KCl were recorded. Ag/AgCl electrodes were employed to characterize the I-V responses.

The pore density [7,8] of the membrane can be calculated by the Equation (2),

$$G_{\text{membrane}} = nG_{\text{individual}} = n\sigma\left(\frac{4l}{\Pi D^2} + \frac{1}{D}\right)^{-1} \tag{2}$$

where G_{membrane}, $G_{\text{individual}}$, n, σ, l and D are the conductance of the whole membrane with nanopore array, the conductance of a single nanopore, the number of nanopores, the conductivity of KCl solution, nanopore length (50 nm), and nanopore diameter (1.5 nm), respectively. The resistance of the membrane is calculated to be 21 Ω after

subtracting the resistance of the drilled PEN support (R_{PEN} = 1791 Ω) from the measured resistance of membrane supported by drilled PEN substrate ($R_{PEN} + R_{membrane}$ = 1812 Ω) according to the measured data in Fig. 2B. Therefore, the pore density of the GNM-COO^- membrane calculated based on Equation (2) is determined to be 1.6×10^{12} cm^{-2}. The conductance of a single nanopore ($G_{individual}$) was calculated using a well-established model for nanofluidic transport, which considers both the pore geometry and the ionic conductivity of the electrolyte.

Molecular dynamics simulations

To investigate the ion transport properties across the GNM-COO^- membrane at the molecular level, we conducted MD simulation through LAMMPS package [9]. The systems mainly consist of single pore GNM-COO^- with different charge densities, K^+ ions, Cl^- ions, and water molecules (Fig. 2D). We varied the pore charge of the GNM-COO^- by changing the type and quantity of oxygen-containing functional groups at the edge of the pores. Specifically, in GNM-xCOO^- membranes (2-COO^-, 4-COO^-, 6-COO^-), the number of carboxylic groups is 2, 4 and 6; the number of hydroxyl groups is 2, 2 and 0; the number of epoxy group is 2, 0 and 0, respectively. In ion diffusion simulation, the concentrations of KCl solution at the two sides of the GNM-COO^- membrane are 500 mM and 10 mM, with a total number of 21980 for water molecules. Additionally, we introduced counter ions to balance the charge of the pores. Two graphene walls with applied force were utilized to control the pressure of the system. We constructed a vacuum layer (3 nm in thickness) outside the wall to eliminate the influence of periodicity boundary in z direction, while the other two boundaries are still periodic. All the MD simulations in this work were carried out in the NVT ensemble with a temperature of 300 K. The time step for all MD simulations was set as 2 fs. The PMF values of K^+ and Cl^- migration along the z-axis direction through GNM-COO^- membrane were calculated with umbrella sampling and WHAM method (Table S5). To investigate the current-voltage (I-V) characteristics, additional MD simulations were performed by applying an external electric field across the membrane in the presence of the same concentration gradient (0.5 M/0.01 M KCl). The resulting ion fluxes under various electric field strengths were used to correlate with the experimental I–V measurements.

Poisson−Nernst−Planck (PNP) simulations

The transport of K^+ ions and Cl^- ions through the GNM-COO^- membrane was simulated by PNP equations[7,10]. The PNP model was based on Nernst-Planck theory, which characterizes ion diffusion, and the Poisson equation, which describes the electrostatic potential.

In a steady state, the Nernst-Planck equation is articulated as:

$$\vec{\nabla} \cdot \left(D_i \nabla c_i + \frac{z_i D_i F c_i}{RT} \nabla \phi \right) = 0 \quad (3)$$

here i denotes both cation and anion. D_i represents the diffusivity of the chemical species, R is the universal gas constant, and T denotes the room temperature. For our simulation, the diffusivity values for the K^+, Cl^- ions are 1.96×10^{-9} m^2 s^{-1} and 6.77 ×

10^{-10} m^2 s^{-1} respectively.

The electric potential is governed by the Poisson's equation:

$$-\nabla \cdot (\varepsilon_r \varepsilon_0 \nabla \phi) = F \sum_i z_i c_i \quad (4)$$

where ε_0, ε_r, and F are the permittivity of vacuum (8.85 × 10^{-12} F m^{-1}), relative permittivity of the solution (with a value of 78 in our study), and Faraday constant respectively. z and c define the valence and concentration of ions separately.

The ionic flux, J_i, can be obtained by

$$J_i = D_i \nabla c_i + \frac{z_i D_i F c_i}{RT} \nabla \phi \quad (5)$$

The ion current can be calculated from the cationic and anionic flux across the nanochannel's cross-sectional area by

$$I_i = \iint J_i ds \quad (6)$$

For our 2D simulation model, the dimensions of two reservoirs, SWNT, and GNM-COO^- channels, are 500 nm × 500 nm, 50 nm × 30 nm and 1.5 nm × 0.34 nm separately. The pore size of the GNM-COO^- membrane we used in the PNP simulation is 1.5 nm, which was determined to be the optimal pore size after extensive experiments. The surface charge density of the nanoporous graphene and SWNT have been assigned values of −2.4 × 10^{-3} C m^{-2} and −2.4 × 10^{-4} C m^{-2} on their side walls, respectively. Regarding boundary conditions, the low and high concentration are designated to the reservoir's bottom and top boundaries separately. Similarly, voltage bias and ground voltage are also applied onto bottom and top boundary, respectively. Applying a positive voltage on the nanoporous graphene to SWNT side is specified as a positive bias, while applying a positive voltage on the SWNT to nanoporous graphene side is specified as a negative bias.

Supplementary Figures

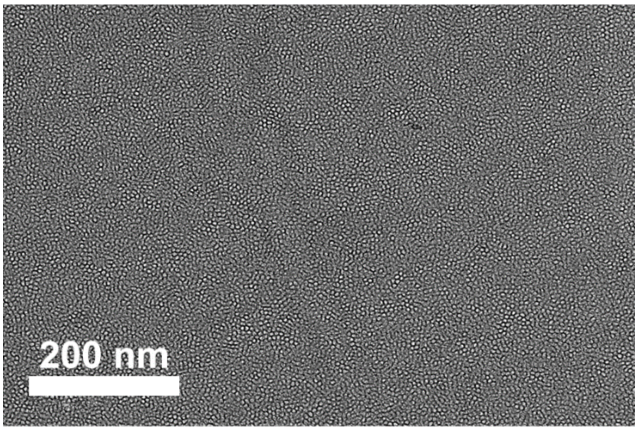

Figure S1 TEM image of the meso-SiO_2 membrane.

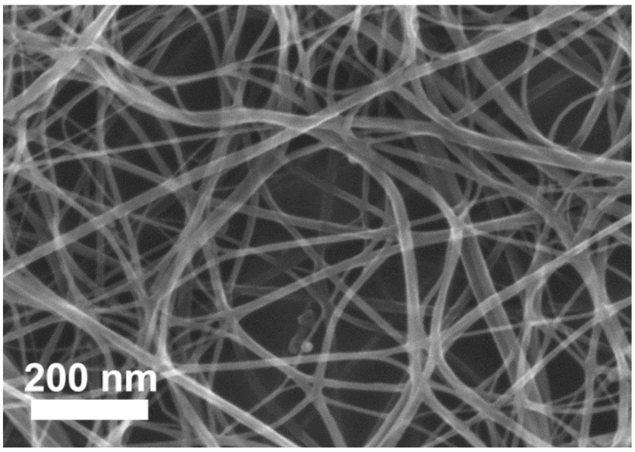

Figure S2 SEM image of the GNM-COO^- membrane.

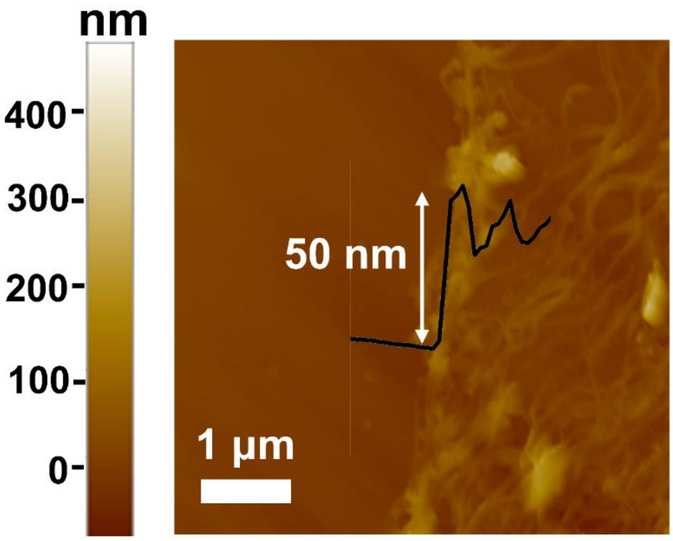

Figure S3 AFM image and height profile of the GNM-COO⁻ membrane.

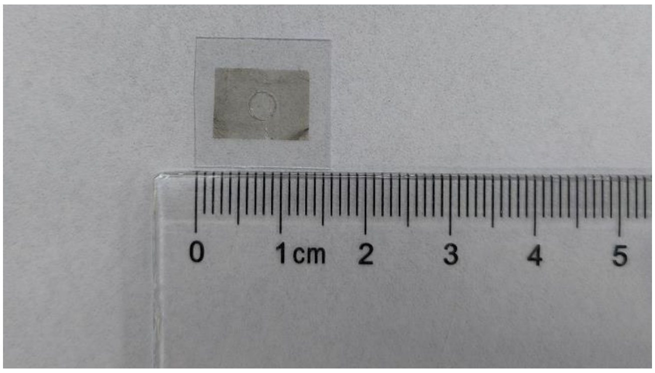

Figure S4 Photograph of the freestanding centimeter-scale GNM-COO⁻ membrane.

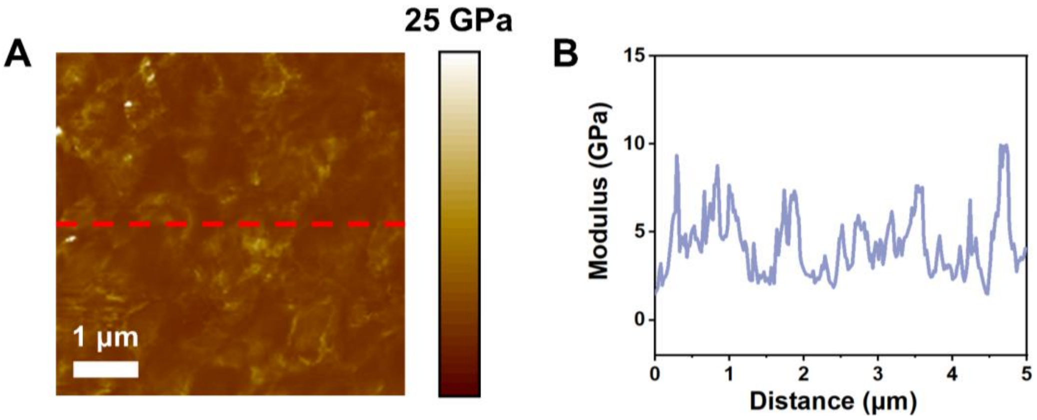

Figure S5 (A) Modulus map and (B) modulus distribution of the GNM-COO^{-} membrane.

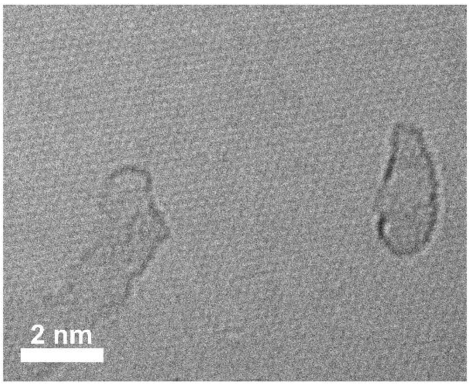

Figure S6 Aberration-corrected TEM image of graphene.

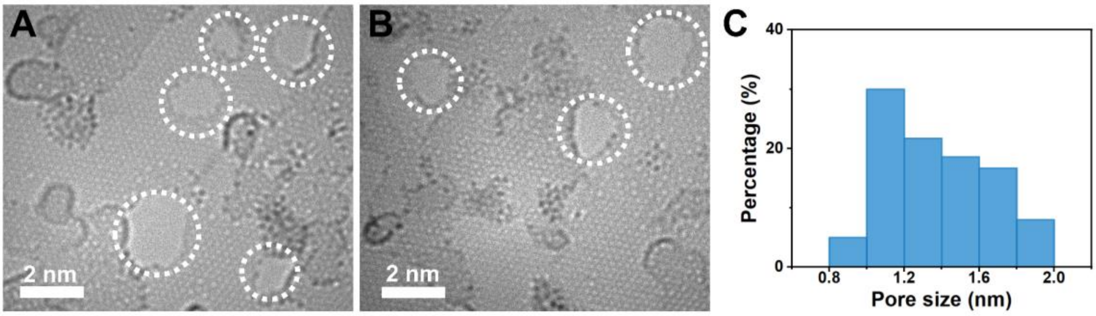

Figure S7 (A, B) Aberration-corrected TEM image and (C) pore size distributions of GNM-COO⁻ membrane prepared by O_2 plasma etching time of 10 s. (A) and (B) represent two samples produced with the same conditions.

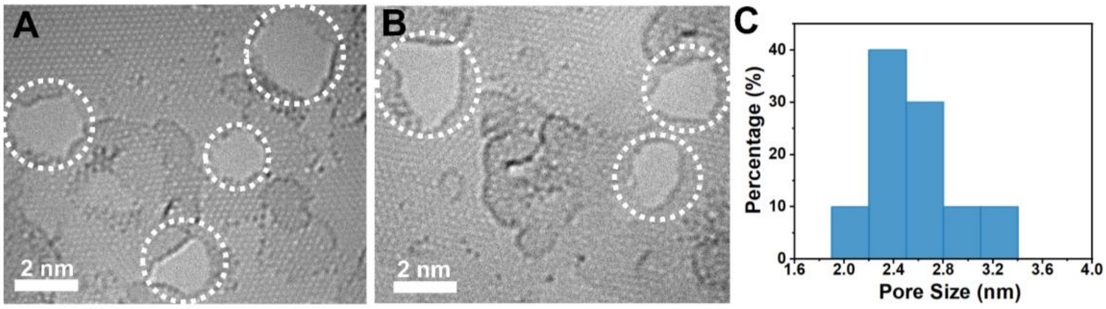

Figure S8 (A, B) Aberration-corrected TEM image and (C) pore size distributions of GNM-COO⁻ membrane prepared by O_2 plasma etching time of 20 s. (A) and (B) represent two samples produced with the same conditions.

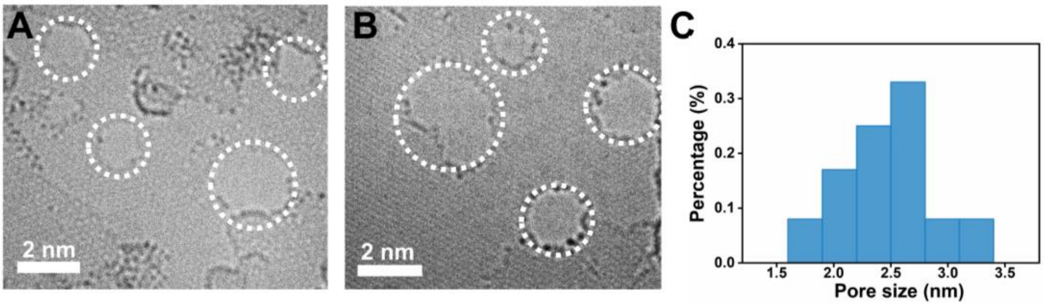

Figure S9 (A, B) Aberration-corrected TEM image and (C) pore size distributions of GNM-COO^- membrane after Hummers' treatment prepared by O_2 plasma etching time of 20 s. (A) and (B) represent two samples produced with the same conditions.

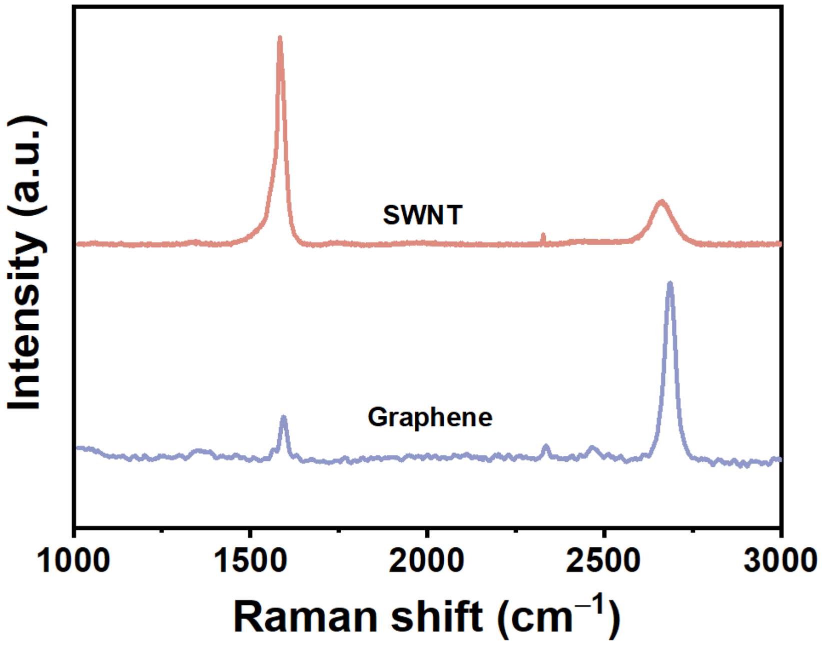

Figure S10 Raman spectra of graphene and SWNT membranes.

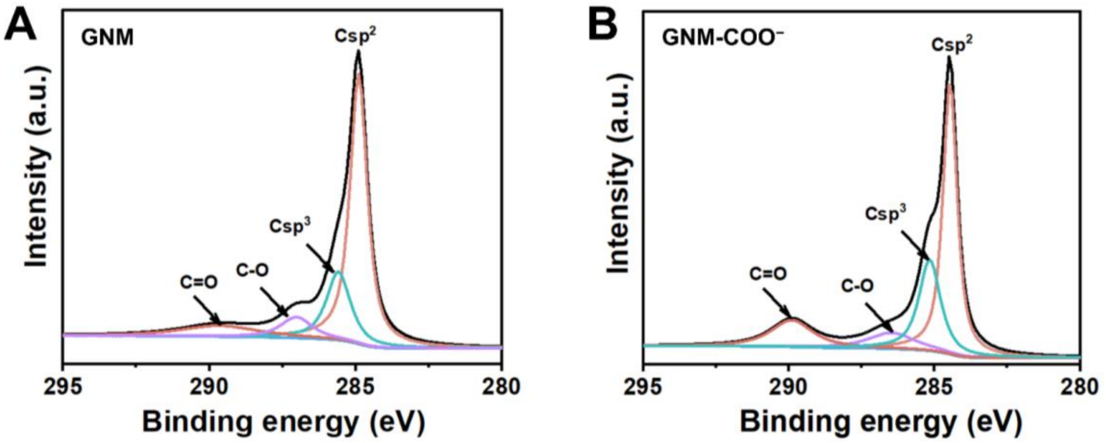

Figure S11 XPS C1s core of (A) GNM and (B) GNM-COO^- membranes. After chemical modification, the peaks correspond to C=O functionalities for the GNM-COO^- membrane exhibit an obvious increase compared with GNM membrane.

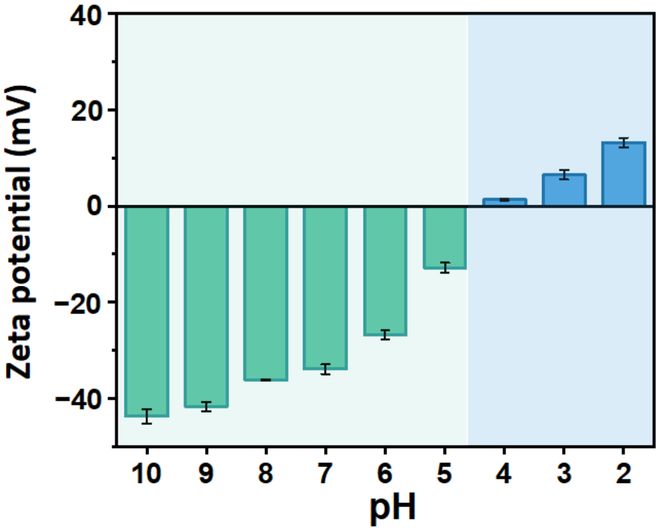

Figure S12 Zeta potential of the GNM-COO^- membrane as a function of pH. Error bars represent the s.d. with three parallel experiments.

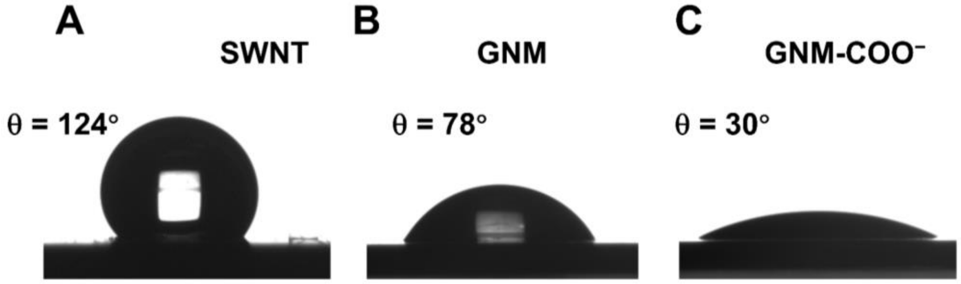

Figure S13 Contact angle of the (A) SWNT, (B) GNM and GNM-COO⁻ membranes.

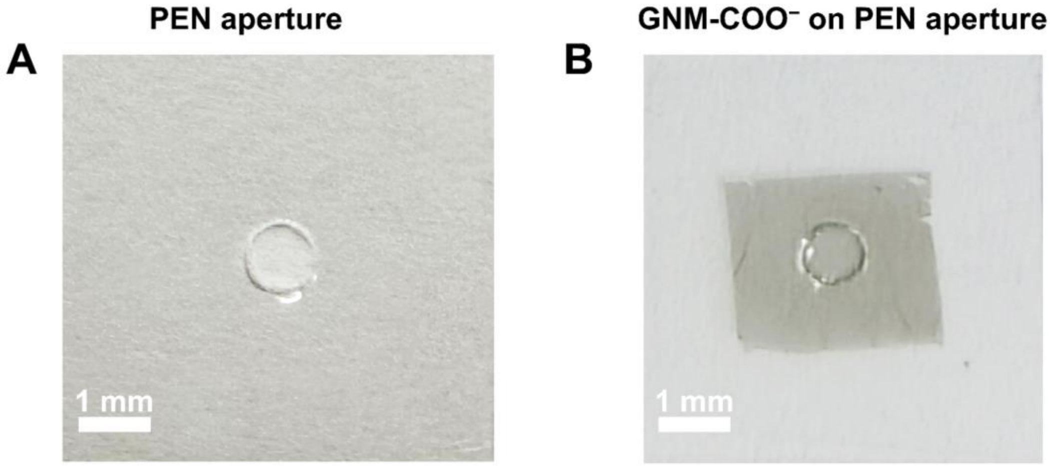

Figure S14 Optical images of (A) a PEN support with an aperture of 0.78 mm^{-2} and (B) a layer of GNM-COO⁻ covered on a PEN aperture.

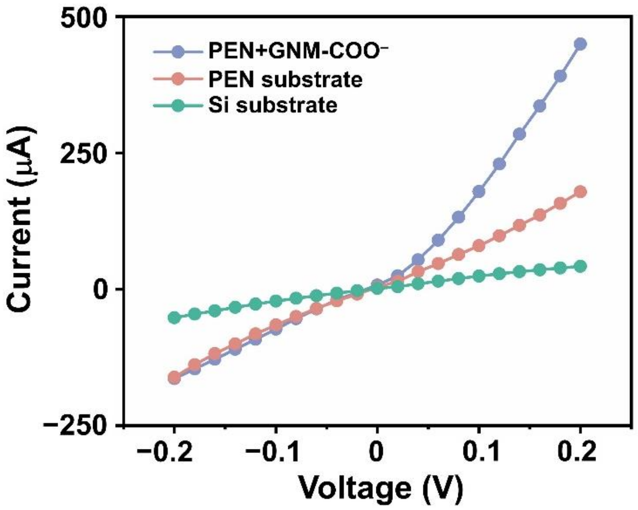

Figure S15 I-V curves of the PEN+GNM-COO^- membrane, PEN substrate, and Si substrate measured in 0.5 M KCl.

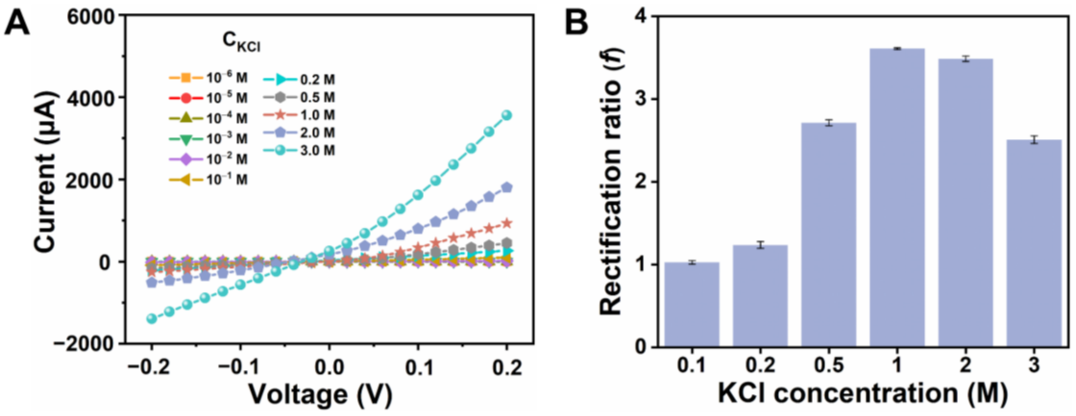

Figure S16 (A) I-V curves of the GNM-COO^- membrane at different KCl concentrations. (B) Rectification ratio of the GNM-COO^- membrane at different KCl concentrations.

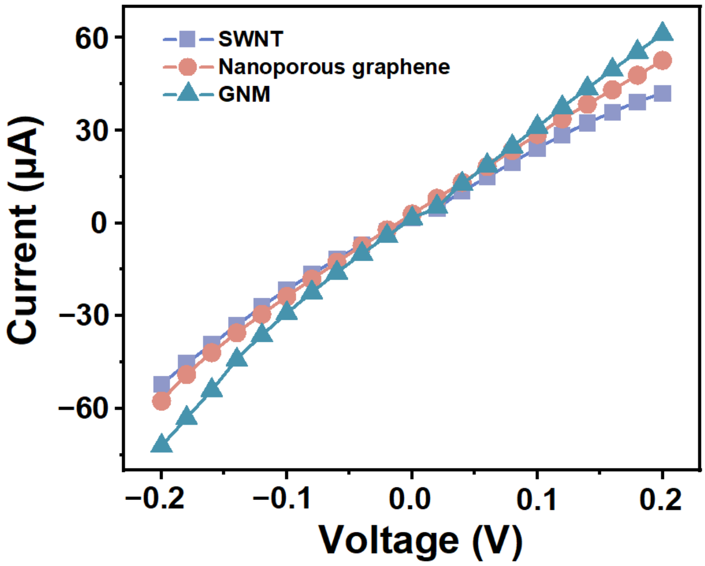

Figure S17 I-V curves of the SWNT, nanoporous graphene and GNM membrane at 1 M KCl concentration.

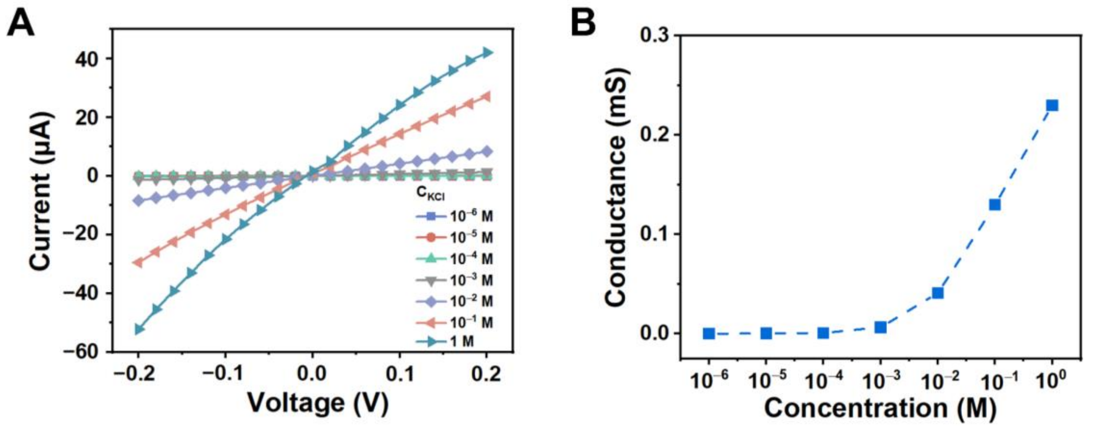

Figure S18 (A) I-V curves of the SWNT membrane at different KCl concentrations. (B) Ionic conductance of the SWNT membrane as a function of electrolyte (KCl) concentration.

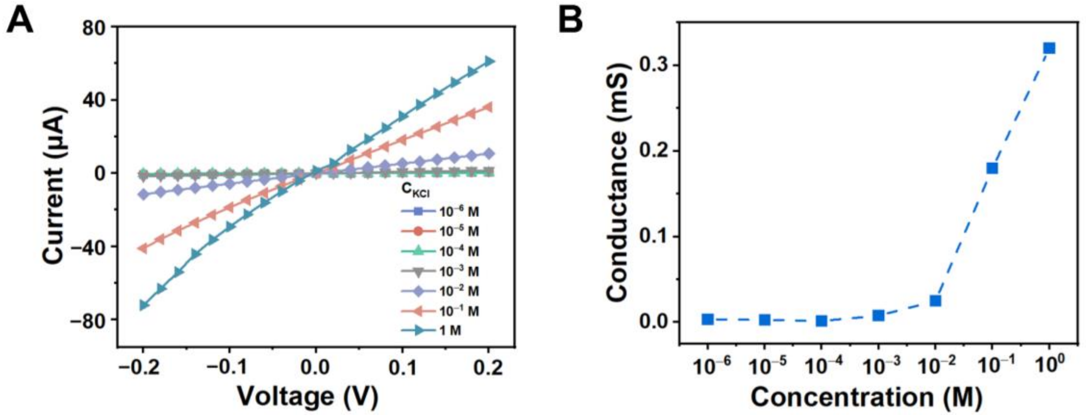

Figure S19 (A) I-V curves of the GNM membrane at different KCl concentrations. (B) Ionic conductance of the GNM membrane as a function of KCl concentration.

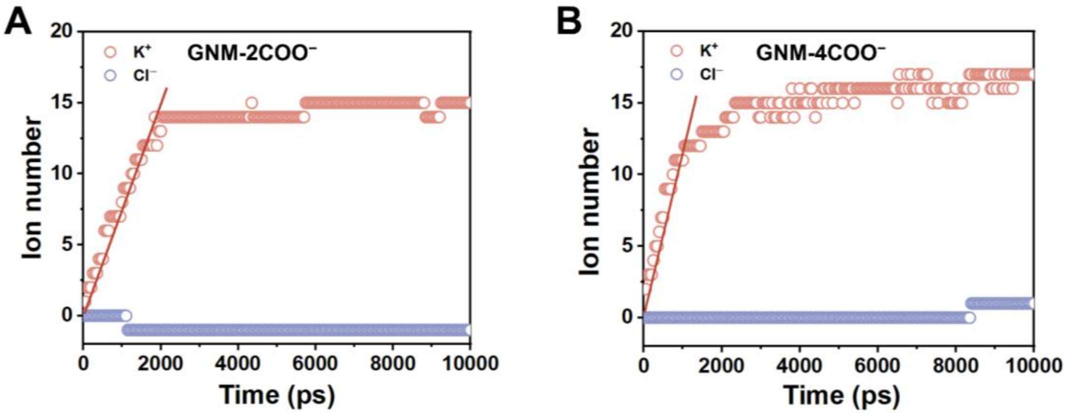

Figure S20 Numbers of hydrated K^+ and Cl^- ions transferred across the (A) GNM-$2COO^-$ and (B) GNM-$4COO^-$ membranes in 0.5 M/0.01 M KCl electrolyte plotted as functions of simulation time.

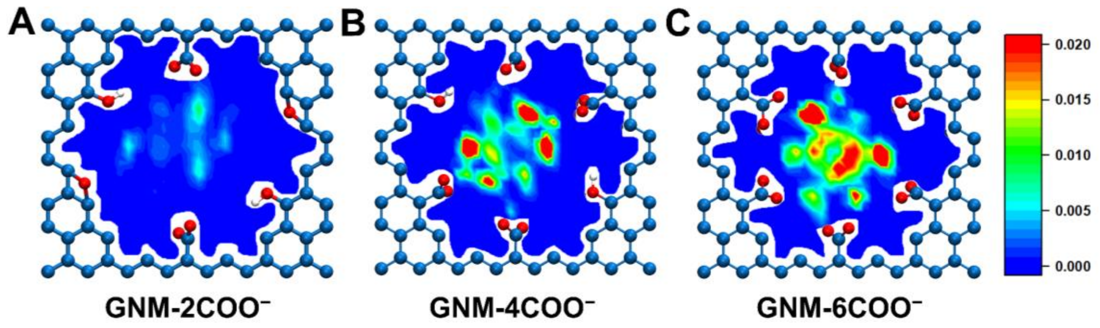

Figure S21 Density distribution profiles of hydrated K^+ in xy-planes for GNM-xCOO⁻ membrane under a concentration gradient of 50 (0.5 M/0.01 M KCl).

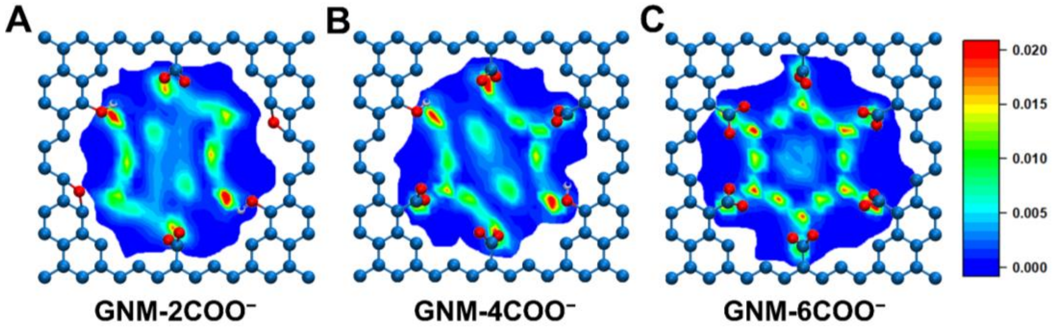

Figure S22 Density distribution profiles of O atoms of water in xy-planes for GNM-xCOO⁻ membrane under a concentration gradient of 50 (0.5 M/0.01 M KCl).

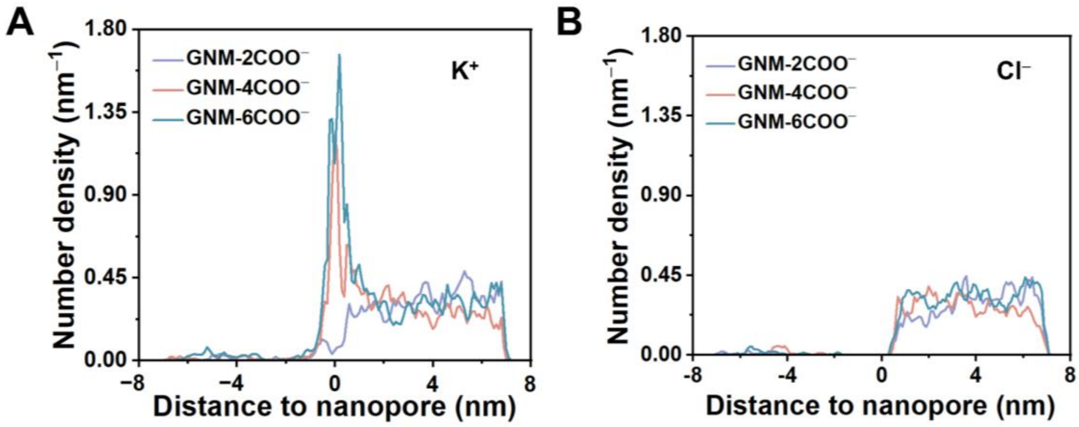

Figure S23 MD simulation of the concentration distributions of (A) K^+ and (B) Cl^- as a function of vertical distance from the pore center of GNM-xCOO⁻ membrane under KCl concentration gradient of 50 (0.5 M/0.01 M).

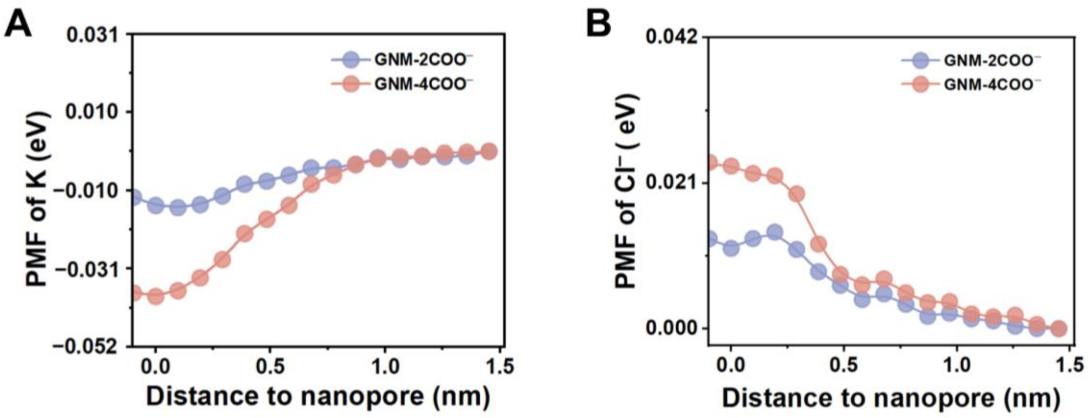

Figure S24 PMF profiles for (A) hydrated K^+ and (B) hydrated Cl^- transport through the GNM-xCOO⁻ membranes under concentration gradient of 0.5 M/0.01 M KCl electrolyte.

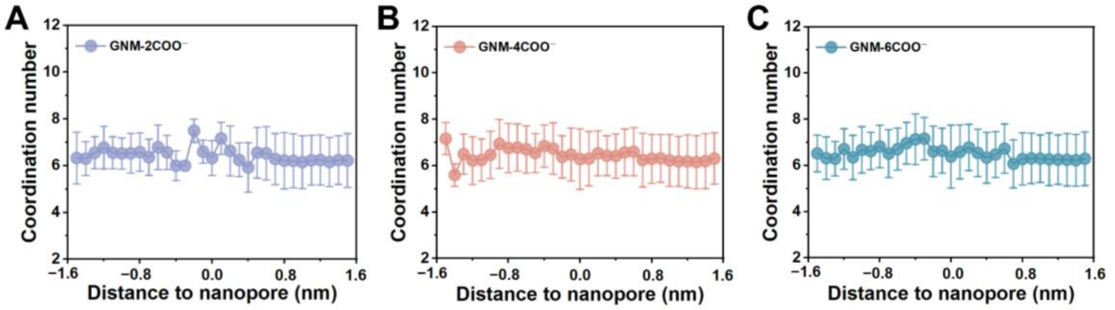

Figure S25 Coordination number of water at the selected locations when hydrated K^+ transport through the (**A**) GNM-2COO⁻, (**B**) GNM-4COO⁻ and (**C**) GNM-6COO⁻ membranes. Error bars represent the s.d. with three parallel experiments.

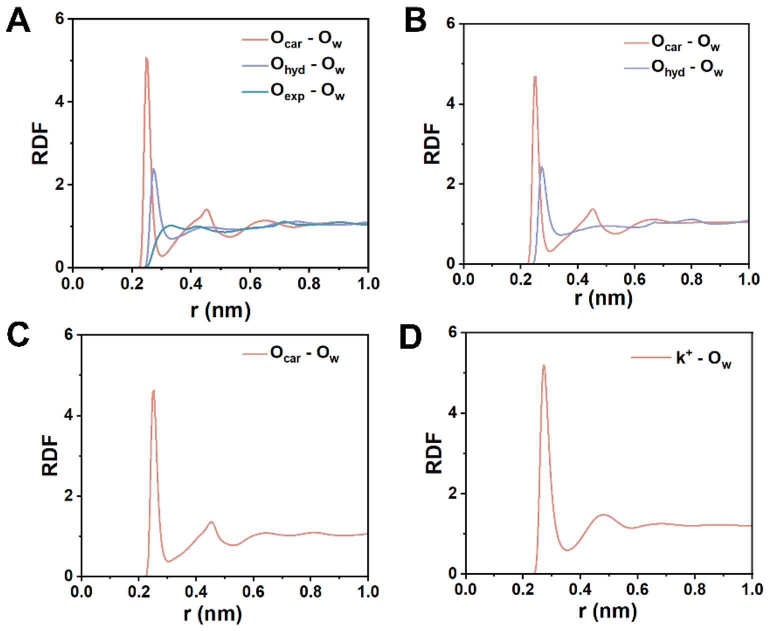

Figure S26 Radial distribution function (RDF) of functional group for (A) GNM-2COO⁻, (B) GNM-4COO⁻, (C) GNM-6COO⁻ membranes and (D) hydrated K^+.

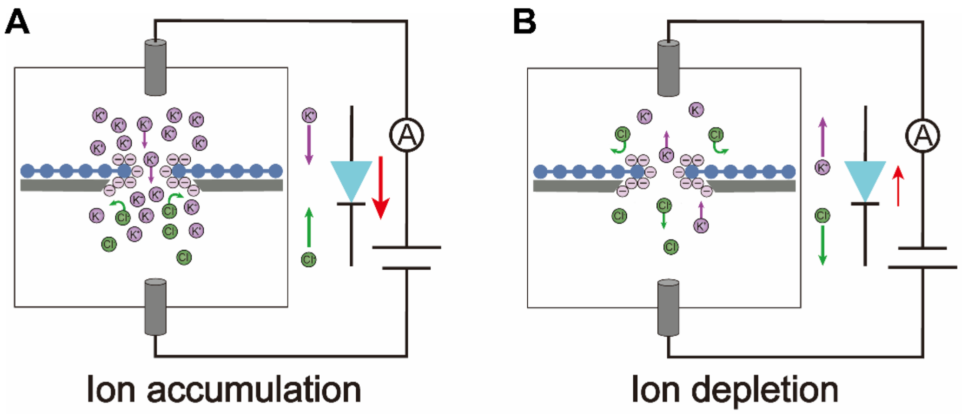

Figure S27 Schematic of the ions rectification mechanism in the GNM-COO^-. (A) Enhanced ion conductivity was observed at positive voltage; (B) low ion conductivity was observed at negative voltage.

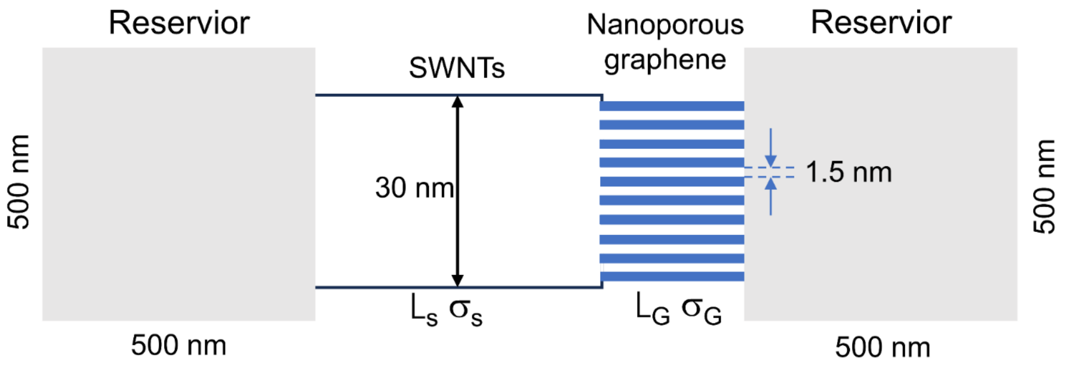

Figure S28 The simulated 2D domain for PNP model. Two KCl electrolyte reservoirs are connected by a two-segment nanochannel composed of the nanoporous graphene and SWNT. In all the simulations, the concentration and potential of the central pore are used to calculate the current.

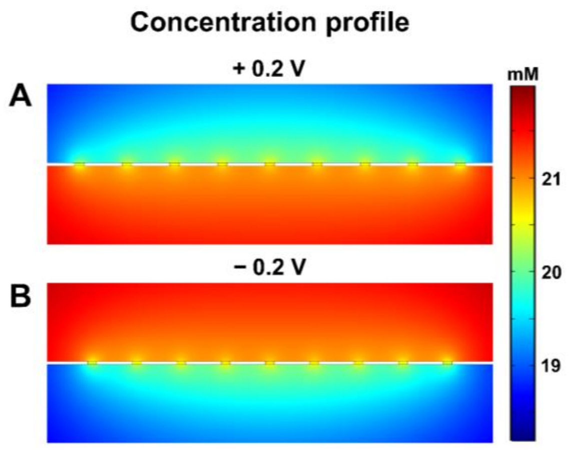

Figure S29 Simulated ions concentration profiles in the nanoporous graphene membrane with applied voltage bias of (A) + 0.2 V and (B) − 0.2 V.

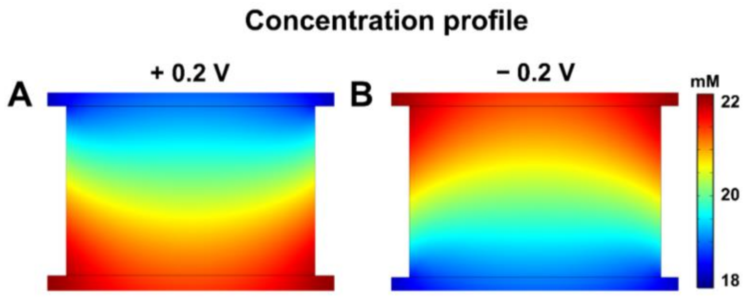

Figure S30 Simulated ions concentration profiles in the SWNT membrane with applied voltage bias of (A) + 0.2 V and (B) − 0.2 V.

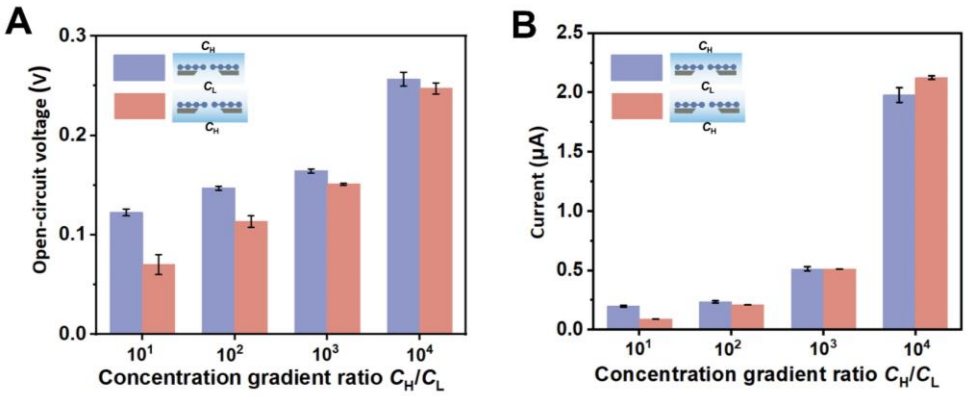

Figure S31 The (A) V_{oc} and (B) I_{sc} of the GNM-COO⁻ at a series of KCl concentration gradients in two concentration configurations.

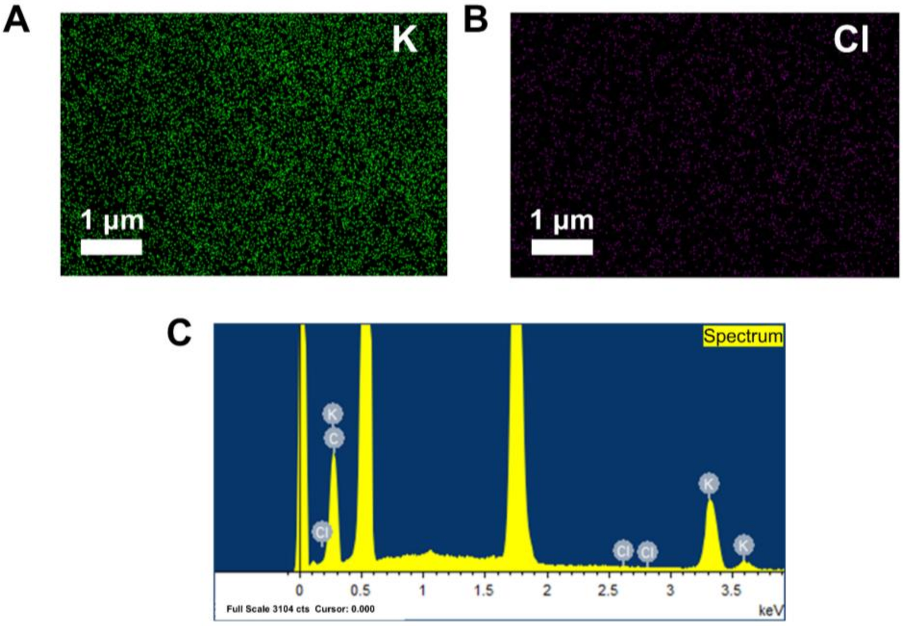

Figure S32 EDX analysis of the GNM-COO⁻ membrane: (A) K and (B) Cl elemental maps, and (C) the corresponding EDX spectrum.

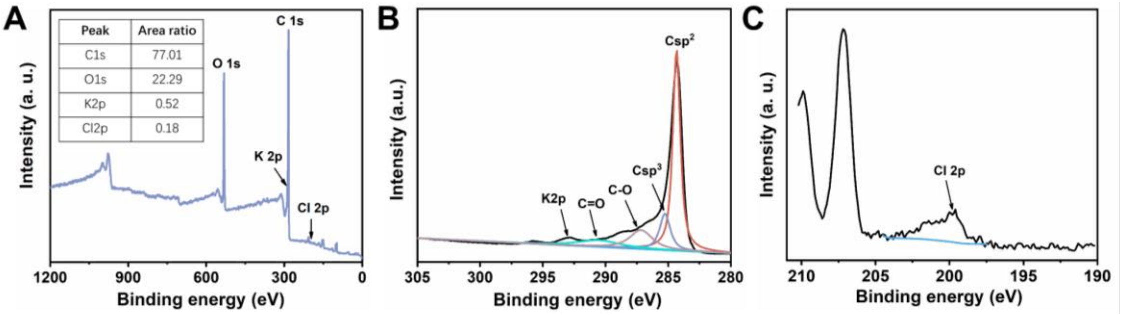

Figure S33 X-ray photoelectron spectroscopy (XPS) of the GNM–COO^{-} membrane: (A) full survey spectrum, (B) high resolution spectra of C1s and K2p, and (C) high resolution Cl2p spectrum.

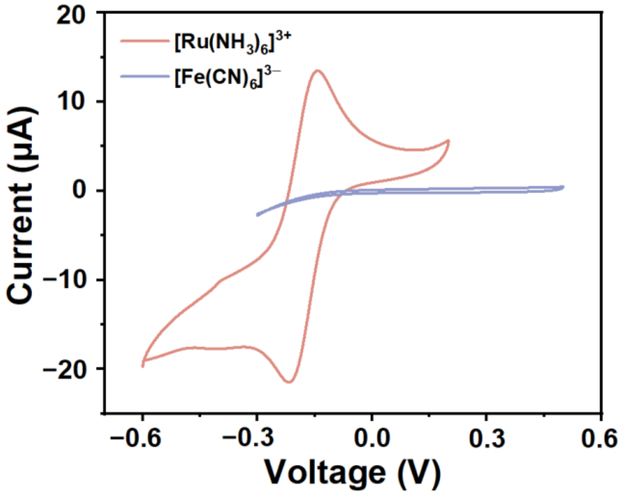

Figure S34 CV curves of the GNM-COO^{-}. ($[Ru(NH_3)_6]^{3+}$ serves as a cationic electroactive probe (red curve); $[Fe(CN)_6]^{3-}$ acts as an anionic electroactive probe (blue curve).

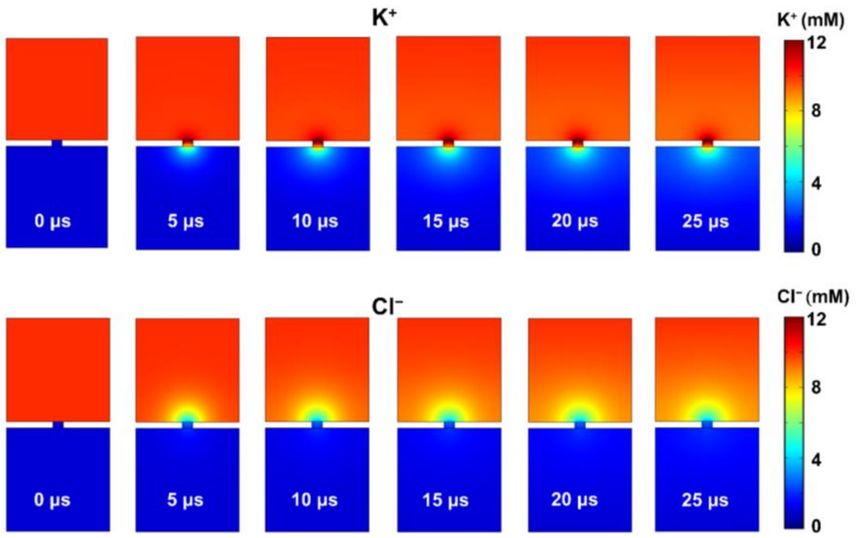

Figure S35 Calculated steady-state concentration distribution of (A) hydrated K^+ and (B) hydrated Cl^- near the GNM-COO⁻ under a salt gradient of 0.5 M/0.01 M KCl, respectively.

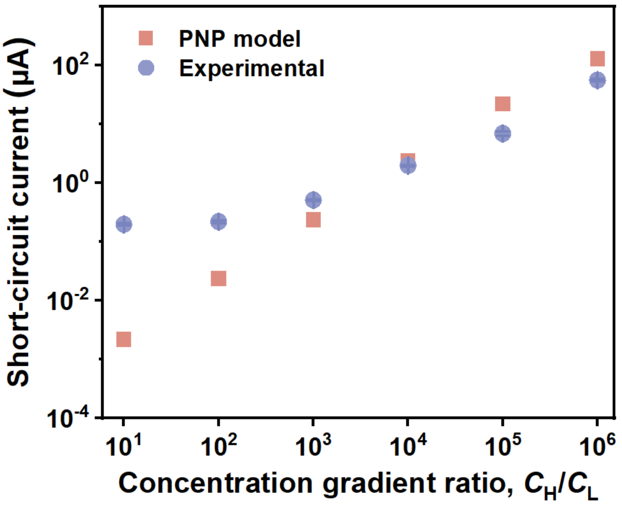

Figure S36 Comparison of the experimental I_{SC} of the GNM-COO⁻ with the simulated value. Error bars represent the s.d. with three parallel experiments.

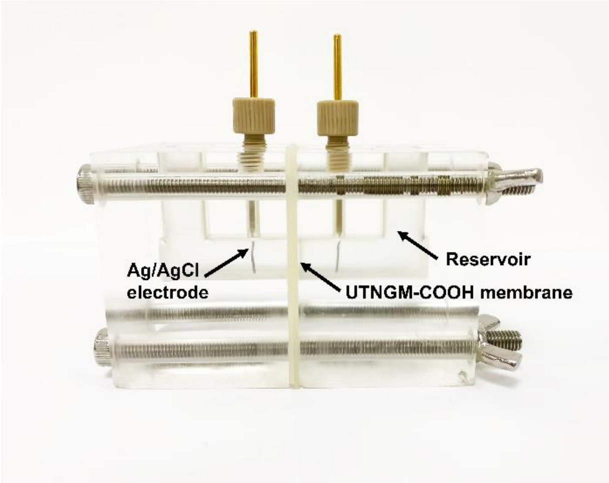

Figure S37 Optical image of the electrochemical device. The GNM-COO⁻ membrane on a Si support with an aperture of 0.03 mm^2 was sandwiched between two reservoirs filled with seawater/river water in each side. Ag/AgCl electrodes were employed to characterize the current-voltage response.

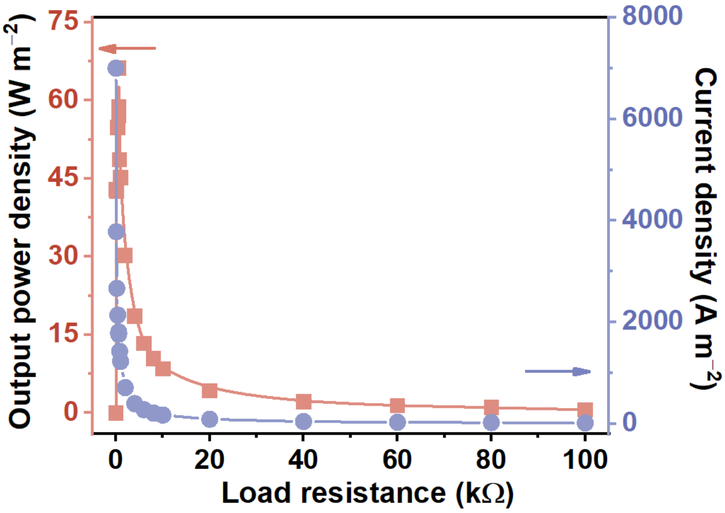

Figure S38 The output power density and current density of the GNM-COO⁻ *versus* load resistance at a salt concentration gradient of 10 (1 M/0.1 M KCl).

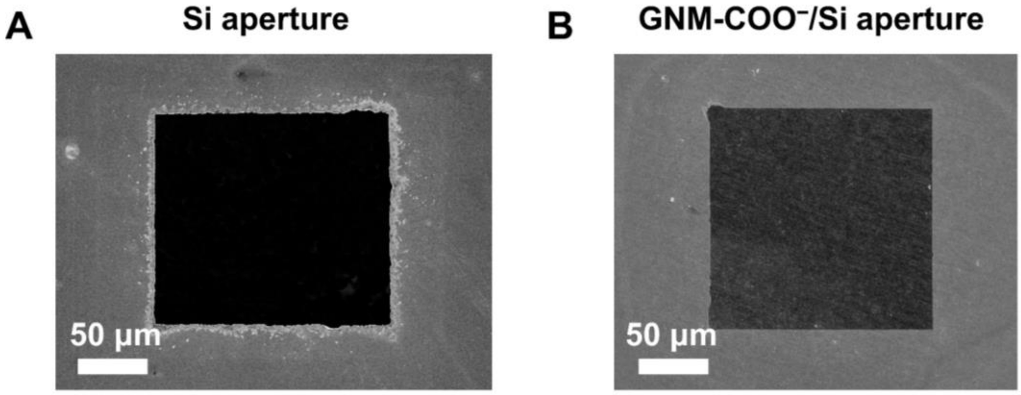

Figure S39 SEM images of (A) a Si aperture with an area of 0.03 mm^{-2} and (B) a layer of GNM-COO^- covered on a Si aperture.

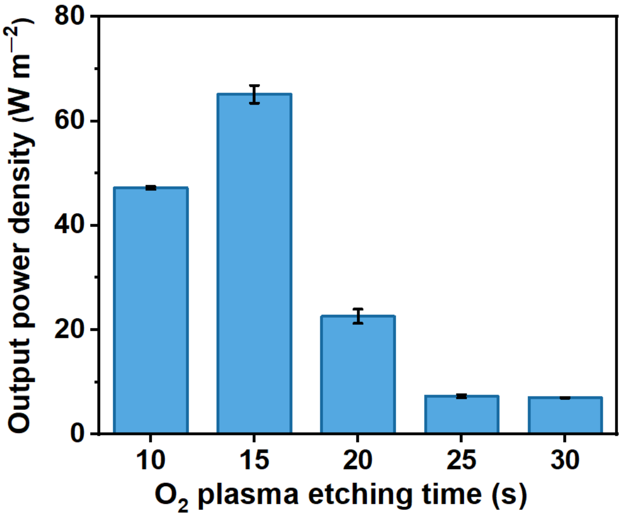

Figure S40 The output power density of the GNM-COO^- prepared with different O_2 plasma etching time. Error bars represent the s.d. with three parallel experiments.

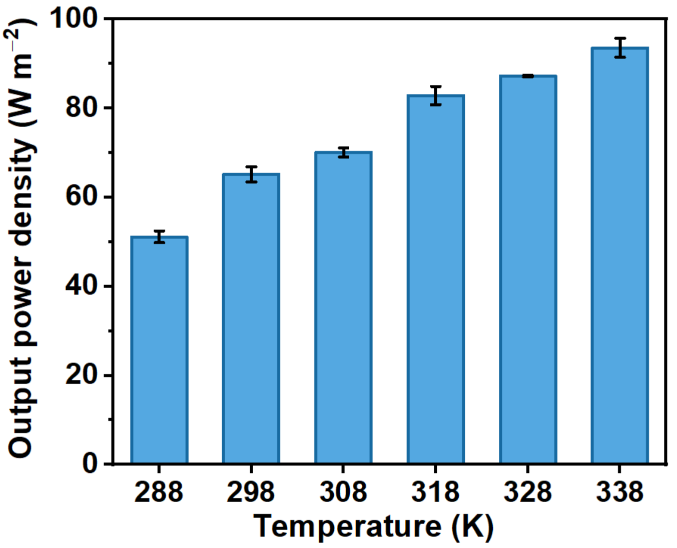

Figure S41 The output power density of the GNM-COO⁻ at different temperatures. Error bars represent the s.d. with three parallel experiments.

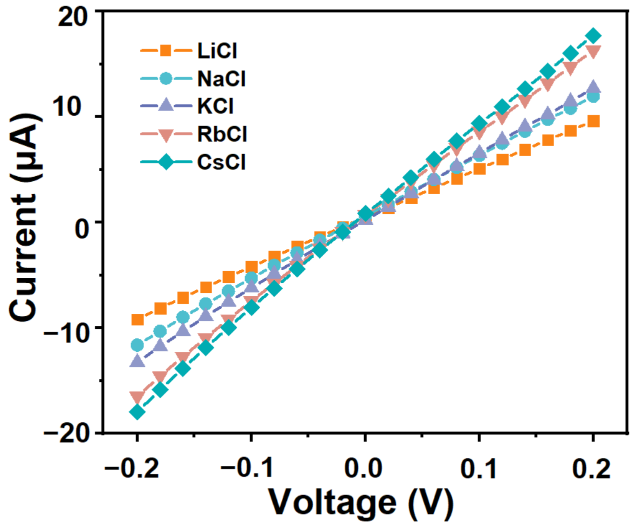

Figure S42 I-V curves of the GNM-COO⁻ at 10^{-3} M LiCl, NaCl, KCl, RbCl and CsCl electrolytes.

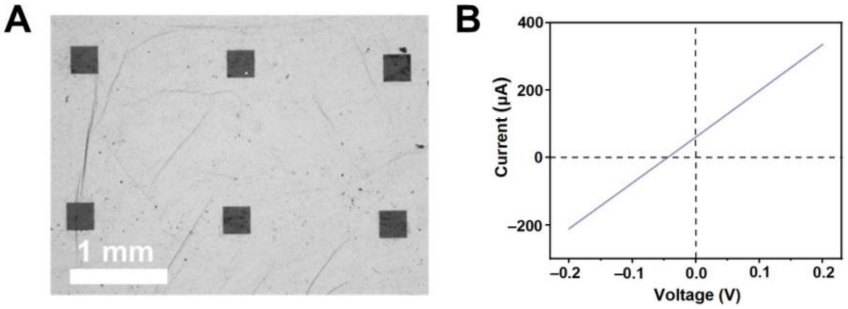

Figure S43 (A) Digital photograph of a representative centimeter-sized GNM-COO^- membrane on a porous 1.3×1.3 cm^2 Si support (10×10 pores with 175 μm side length and 1 mm interpore distance). (B) Current-voltage curve of the GNM-COO^- with an effective test area of 3 mm^{-2} at 0.5 M/0.01 M NaCl gradient.

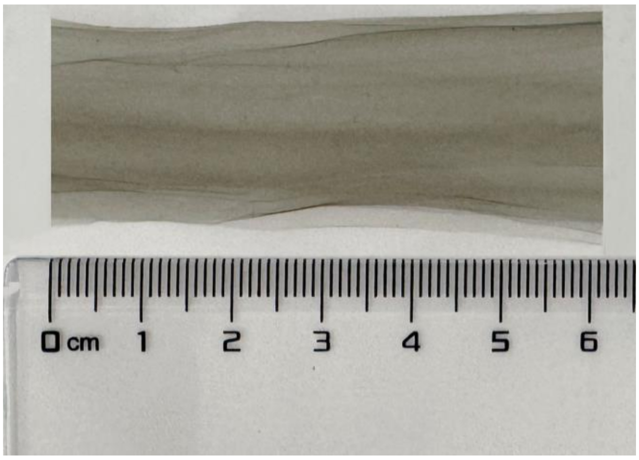

Figure S44 Photograph of a large area GNM-COO^- membrane.

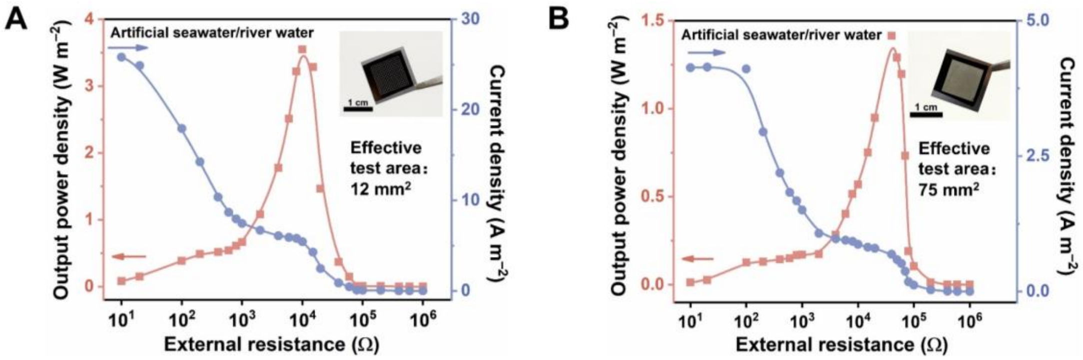

Figure S45 The output power density and current density of the GNM-COO$^-$ membrane with an effective test area of (A) 12 mm^2 and (B) 75 mm^2 at 0.5 M/0.01 M NaCl gradient. Inset: Optical image of a representative centimeter-sized GNM-COO$^-$ membrane on a porous 1.3×1.3 cm^2 Si support.

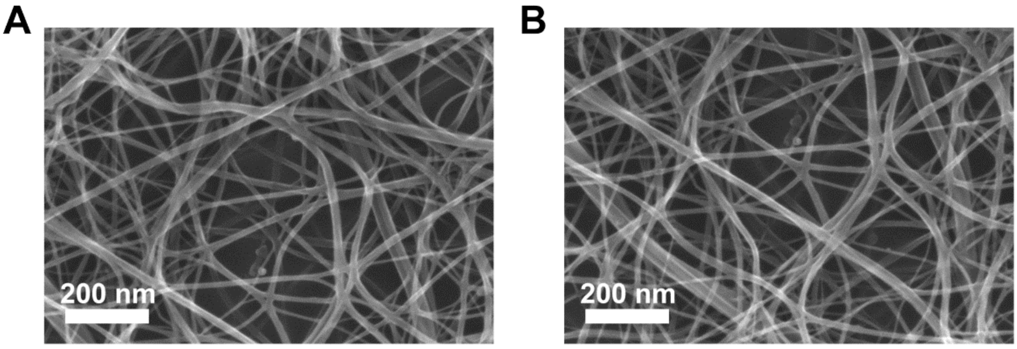

Figure S46 SEM images of GNM-COO$^-$ (A) before and (B) after osmotic energy stability test.

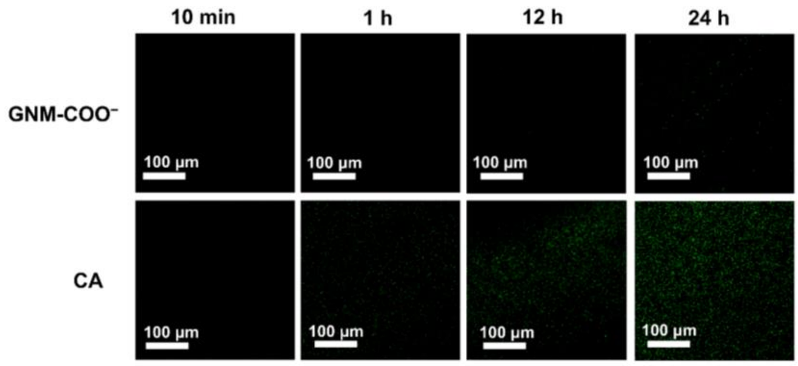

Figure S47 Confocal microscopy images of *R. palustris* cells attached to the GNM-COO^- membrane and CA membrane at different test time periods.

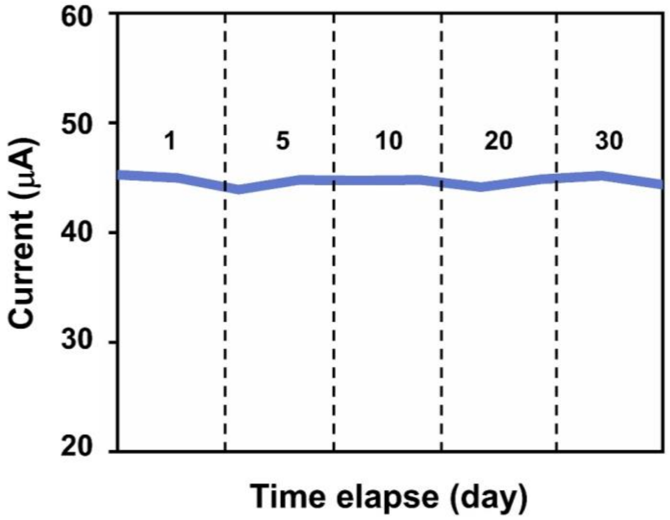

Figure S48 Current-time curve of the GNM-COO^- membrane in artificial seawater/river (0.5 M NaCl/0.01 M NaCl, $MgCl_2$, $CaCl_2$ and *R. palustris*) water without electrolyte replenishment. The R_L is fixed at 2 kΩ.

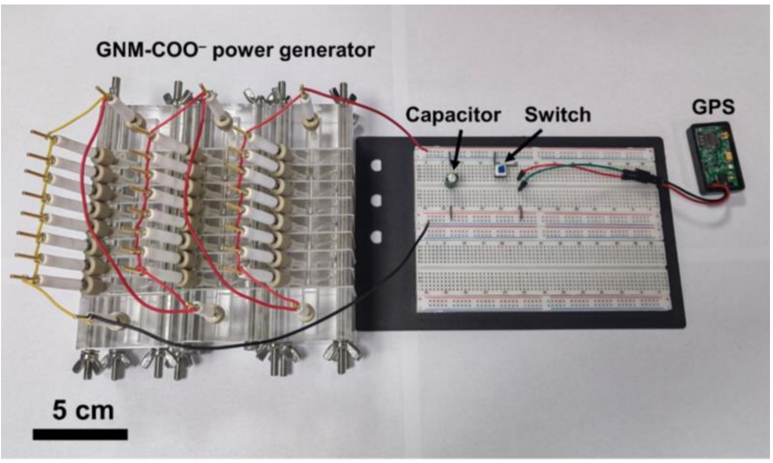

Figure S49 Schematic of the circuit design and working demonstration of the self-powered GPS electronic device. The circuit primarily consists of four elements: GNM-COO^- power generator, capacitor, switch and GPS electronic device.

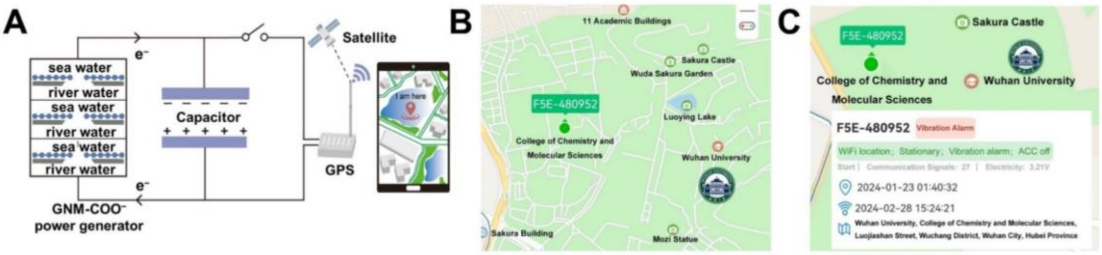

Figure S50 (A) Schematic of a home-made self-powered positioning system; (B) A smart phone interface showing the successfully located position of the lab building in Wuhan University with the GPS. (C) A smartphone interface showing the specific location information of the lab building of College of Chemistry and Molecular Sciences in Wuhan University with the GPS.

Table S1. The open-circuit potential, redox potential, and diffusion potential of the GNM-COO^- at different KCl concentration gradients.

Concentration gradient (M/M)	$10^{-6}/10^{-5}$	$10^{-6}/10^{-4}$	$10^{-6}/10^{-3}$	$10^{-6}/10^{-2}$	$10^{-6}/10^{-1}$	$10^{-6}/1$	0.01/0.5
Open-circuit potential (mV)	84	138	170	203	425	509	130
Redox potential (mV)	57	65	68	70	200	230	45
Diffusion potential (mV)	27	73	102	133	225	279	85

Table S2. Ionic species and their hydrated ionic radii and bulk diffusion coefficient.

**Ionic species**	**Hydrated ionic radii (nm)**	**Diffusion coefficient (cm^2 s^{-1})**
Li^+	0.382	1.00×10^{-5}
Na^+	0.358	1.33×10^{-5}
K^+	0.331	1.83×10^{-5}
Ru^+	0.329	1.98×10^{-5}
Cs^+	0.329	1.99×10^{-5}
Mg^{2+}	0.428	0.71×10^{-5}
Ca^{2+}	0.412	0.97×10^{-5}
Cl^-	0.332	2.03×10^{-5}

Table S3. Energy conversion performance, thickness and load resistance of the GNM-COO^- compared with the state-of-art membranes at a salt concentration gradient of 50.

Membrane	Concentration gradient	Thickness (μm)	Power density (W m^{-2})	Load resistance (kΩ)
MOFs/PAA [11]	50	52.8	1.6	20
MOFs/PSS [12]	50	85	2.87	302
AAO [13]	50	99.2	3.15	28
MesoC/MacroA [14]	50	64.2	3.46	10
Vermiculite [15]	50	0.001	4.5	22
MXBN [16]	50	10	6.2	170
AAO/GO [17]	50	15	3.73	91
PPy [18]	50	1.5	26.22	1.8
BP/GO [19]	50	8	4.7	250
ICM-8 [20]	50	0.1	16.72	27
MOFs/AAO [21]	50	0.5	2.04	40
BCP/PEI [22]	50	0.1	22.4	60
COF [9]	50	0.1	43.2	3
ZnTPP–COF [7]	50	0.001	135.8	500
Carbon membranes [8]	50	0.002	67	390
This Work	50	0.05	175.1	2

Table S4. Summary of the state-of-art osmotic power density of large-area nanoporous membranes at a salt concentration gradient of 50 and other biomimetic ion channels.

Membrane	Test area	Power density	Refs
SPEEK/PES	0.2 mm^2	16.5 (LiBr) W m^{-2}	[23]
ZnTPP-COF	0.25 mm^2	14.63 W m^{-2}	[7]
Polypyrrole	3.14 mm^2	0.087 W m^{-2}	[24]
GO	0.8 mm^2	0.77 W m^{-2}	[25]
MOFs/PAA	0.875 mm^2	1.6 W m^{-2}	[12]
ABN	3.14 mm^2	0.6 W m^{-2}	[26]
Polyacrylamide hydrogels	28 mm^2	0.37 W m^{-2}	[27]
BNNTs	2.1 nm	12 KW m^{-2}	[28-30]
GNM-COO^-	3 mm^2	5.3 W m^{-2}	This work

Table S5 Atomistic coordinates for the GNM-xCOO^-

Atoms number	Atoms list	Change	x	y	z
1	C2	0.00	24.60	22.70	107.00
2	C2	0.00	25.80	24.90	107.00
3	C2	0.00	24.60	24.10	107.00
4	C2	0.00	27.10	22.70	107.00
5	C2	0.00	28.30	24.90	107.00
6	C2	0.00	27.10	24.10	107.00
7	C2	0.00	29.50	22.70	107.00
8	C2	0.00	29.50	24.10	107.00
9	C2	0.00	23.40	29.10	107.00
10	C2	0.00	23.40	26.30	107.00
11	C2	0.00	24.60	27.00	107.00
12	C2	0.00	25.80	29.10	107.00
13	C2	0.00	24.60	28.40	107.00
14	C2	0.00	25.80	26.30	107.00
15	C2	0.00	27.10	27.00	107.00
16	C2	0.00	28.30	29.10	107.00
17	C2	0.00	27.10	28.40	107.00
18	C2	0.00	28.30	26.30	107.00
19	C2	0.00	29.50	27.00	107.00
20	C2	0.00	29.50	28.40	107.00
21	C2	0.00	30.70	24.90	107.00
22	C2	0.00	32.00	22.70	107.00
23	C2	0.00	33.20	24.90	107.00
24	C2	0.00	32.00	24.10	107.00
25	C2	0.00	34.40	22.70	107.00
26	C2	0.00	35.70	24.90	107.00
27	C2	0.00	34.40	24.10	107.00
28	C2	0.00	36.90	22.70	107.00
29	C2	0.00	36.90	24.10	107.00
30	C2	0.00	33.20	26.30	107.00
31	C2	0.00	32.00	27.00	107.00
32	C2	0.00	36.90	27.00	107.00
33	C2	0.00	30.70	26.30	107.00
34	C2	0.00	35.70	26.30	107.00
35	C2	0.00	38.10	24.90	107.00
36	C2	0.00	39.40	22.70	107.00
37	C2	0.00	40.60	24.90	107.00
38	C2	0.00	39.40	24.10	107.00
39	C2	0.00	41.80	22.70	107.00
40	C2	0.00	43.00	24.90	107.00
41	C2	0.00	41.80	24.10	107.00
42	C2	0.00	44.30	22.70	107.00
43	C2	0.00	44.30	24.10	107.00

44	C2	0.00	41.80	28.40	107.00
45	C2	0.00	40.60	26.30	107.00
46	C2	0.00	44.30	27.00	107.00
47	C2	0.00	41.80	27.00	107.00
48	C2	0.00	40.60	29.10	107.00
49	C2	0.00	38.10	26.30	107.00
50	C2	0.00	39.40	27.00	107.00
51	C2	0.00	44.30	28.40	107.00
52	C2	0.00	43.00	29.10	107.00
53	C2	0.00	43.00	26.30	107.00
54	C2	0.00	24.60	31.20	107.00
55	C2	0.00	25.80	33.40	107.00
56	C2	0.00	23.40	37.60	107.00
57	C2	0.00	23.40	34.80	107.00
58	C2	0.00	24.60	35.50	107.00
59	C2	0.00	25.80	37.60	107.00
60	C2	0.00	24.60	36.90	107.00
61	C2	0.00	25.80	34.80	107.00
62	C2	0.00	27.10	35.50	107.00
63	C2	0.00	27.10	36.90	107.00
64	C2	0.00	44.30	32.70	107.00
65	C2	0.00	41.80	31.20	107.00
66	C2	0.00	43.00	33.40	107.00
67	C2	0.00	41.80	32.70	107.00
68	C2	0.00	44.30	31.20	107.00
69	C2	0.00	43.00	30.50	107.00
70	C2	0.00	43.00	37.60	107.00
71	C2	0.00	41.80	36.90	107.00
72	C2	0.00	43.00	34.80	107.00
73	C2	0.00	44.30	35.50	107.00
74	C2	0.00	44.30	36.90	107.00
75	C2	0.00	24.60	39.80	107.00
76	C2	0.00	25.80	41.90	107.00
77	C2	0.00	24.60	41.20	107.00
78	C2	0.00	25.80	39.00	107.00
79	C2	0.00	27.10	39.80	107.00
80	C2	0.00	28.30	41.90	107.00
81	C2	0.00	27.10	41.20	107.00
82	C2	0.00	28.30	39.00	107.00
83	C2	0.00	29.50	39.80	107.00
84	C2	0.00	29.50	41.20	107.00
85	C2	0.00	23.40	43.30	107.00
86	C2	0.00	24.60	44.00	107.00
87	C2	0.00	25.80	43.30	107.00
88	C2	0.00	27.10	44.00	107.00
89	C2	0.00	28.30	43.30	107.00

90	C2	0.00	29.50	44.00	107.00
91	C2	0.00	30.70	41.90	107.00
92	C2	0.00	33.20	41.90	107.00
93	C2	0.00	32.00	41.20	107.00
94	C2	0.00	35.70	41.90	107.00
95	C2	0.00	36.90	41.20	107.00
96	C2	0.00	30.70	43.30	107.00
97	C2	0.00	32.00	44.00	107.00
98	C2	0.00	33.20	43.30	107.00
99	C2	0.00	34.40	44.00	107.00
100	C2	0.00	35.70	43.30	107.00
101	C2	0.00	36.90	44.00	107.00
102	C2	0.00	38.10	41.90	107.00
103	C2	0.00	39.40	39.80	107.00
104	C2	0.00	40.60	41.90	107.00
105	C2	0.00	39.40	41.20	107.00
106	C2	0.00	40.60	39.00	107.00
107	C2	0.00	41.80	39.80	107.00
108	C2	0.00	43.00	41.90	107.00
109	C2	0.00	41.80	41.20	107.00
110	C2	0.00	43.00	39.00	107.00
111	C2	0.00	44.30	39.80	107.00
112	C2	0.00	44.30	41.20	107.00
113	C2	0.00	38.10	43.30	107.00
114	C2	0.00	39.40	44.00	107.00
115	C2	0.00	40.60	43.30	107.00
116	C2	0.00	41.80	44.00	107.00
117	C2	0.00	43.00	43.30	107.00
118	C2	0.00	44.30	44.00	107.00
119	C3	-0.10	34.40	27.00	107.00
120	C3	-0.10	34.40	41.20	107.00
121	C4	0.14	28.30	30.50	107.00
122	C4	0.14	27.10	32.70	107.00
123	C4	0.14	40.60	37.60	107.00
124	C4	0.14	41.80	35.50	107.00
125	C5	0.20	28.30	37.60	107.00
126	C5	0.20	40.60	30.50	107.00
127	C6	0.70	34.40	28.30	107.00
128	C6	0.70	34.30	39.80	107.00
129	O7	-0.80	34.20	28.90	106.00
130	O7	-0.80	35.00	39.20	106.00
131	O7	-0.80	34.80	29.00	108.00
132	O7	-0.80	33.90	39.10	108.00
133	O8	-0.64	29.40	36.90	107.00
134	O8	-0.64	39.40	31.30	107.00
135	H9	0.44	29.10	36.00	106.00

136	H9	0.44	39.50	32.20	107.00
137	O10	-0.28	40.80	36.30	106.00
138	O10	-0.28	28.00	31.80	108.00

References

1 Yang Y, Yang X, Zou X, et al., Ultrafine graphene nanomesh with large on/off ratio for high-performance flexible biosensors. *Adv. Funct. Mater.* 2017, **27**: 1604096.

2 Upan J, Youngvises N, Tuantranont A, et al., A simple label-free electrochemical sensor for sensitive detection of alpha-fetoprotein based on specific aptamer immobilized platinum nanoparticles/carboxylated-graphene oxide. *Sci. Rep.* 2021, **11**: 13969.

3 Zhang Z, Bhauriyal P, Sahabudeen H, et al., Cation-selective two-dimensional polyimine membranes for high-performance osmotic energy conversion. *Nat. Commun.* 2022, **13**: 3935.

4 Liu P, Huang C, Guo Y, et al., Ionic-nanotube array membrane generating ultrahigh osmotic energy conversion. *Adv. Mater.* 2025, **37**: 2506913.

5 Liu S H, Zhang D, Fang Y P, et al., Topologically programmed graphene oxide membranes with bioinspired superstructures toward boosting osmotic energy harvesting. *Adv. Funct. Mater.* 2022, **33**: 2211532.

6 Ding L, Zheng M, Xiao D, et al., Bioinspired $Ti_3C_2T_x$ MXene-based ionic diode membrane for high-efficient osmotic energy conversion. *Angew. Chem. Int. Ed.* 2022, **61**: e202206152.

7 Yang J, Tu B, Zhang G, et al., Advancing osmotic power generation by covalent organic framework monolayer. *Nat. Nanotechnol.* 2022, **17**: 622–628.

8 Liu X, He M, Calvani D, et al., Power generation by reverse electrodialysis in a single-layer nanoporous membrane made from core-rim polycyclic aromatic hydrocarbons. *Nat. Nanotechnol.* 2020, **15**: 307–312.

9 Cao L, Chen I C, Chen C, et al., Giant osmotic energy conversion through vertical-aligned ion-permselective nanochannels in covalent organic framework membranes. *J. Am. Chem. Soc.* 2022, **144**: 12400−12409.

10 Zhou S, Xie L, Li X, et al., Interfacial super-assembly of ordered mesoporous carbon-ailica/AAO hybrid membrane with enhanced permselectivity for temperature- and pH-sensitive smart ion transport. *Angew.Chem. Int. Ed.* 2021, **60**: 26167–26176.

11 Wang C, Liu F F, Tan Z, et al., Fabrication of bio-inspired 2D MOFs/PAA hybrid membrane for asymmetric ion transport. *Adv. Funct. Mater.* 2019, **30**: 1908804.

12 Li R, Jiang J, Liu Q, et al., Hybrid nanochannel membrane based on polymer/MOF for high-performance salinity gradient power generation. *Nano Energy* 2018, **53**: 643–649.

13 Xiao T, Zhang Q, Jiang J, et al., pH-Resistant nanofluidic diode membrane for high-performance conversion of salinity gradient into electric energy. *Energy Technol.* 2019, **7**: 1800952.

14 Gao J, Guo W, Feng D, et al., High-performance ionic diode membrane for salinity gradient power generation. *J. Am. Chem. Soc.* 2014, **136**: 12265−12272.

15 Cao L, Wu H, Fan C, et al., Lamellar porous vermiculite membranes for boosting nanofluidic osmotic energy conversion. *J. Mater. Chem. A* 2021, **9**: 14576–14581.

16 Yang G, Liu D, Chen C, et al., Stable $Ti_3C_2T_x$ MXene-boron nitride membranes with low internal resistance for enhanced salinity gradient energy harvesting. *ACS Nano* 2021, **15**: 6594–6603.

17 Zhang L, Zhou S, Xie L, et al., Interfacial super-assembly of T-mode janus porous heterochannels from layered graphene and aluminum oxide array for smart oriented ion transportation. *Small* 2021, **17**: e2100141.

18 Hao J, Bao B, Zhou J, et al., A euryhaline-fish-inspired salinity self-adaptive nanofluidic diode leads to high-performance blue energy harvesters. *Adv. Mater.* 2022, **34**: 2203109.

19 Zhang Z, Zhang P, Yang S, et al., Oxidation promoted osmotic energy conversion in black

phosphorus membranes. *PNAS* 2020, **117**: 13959–13966.

20 Chen W, Dong T, Xiang Y, et al., Ionic crosslinking-induced nanochannels: nanophase separation for ion transport promotion. *Adv. Mater.* 2022, **34**: e2108410.

21 Guo Y, Huang H, Li Z, et al., Sulfonated sub-nanochannels in a robust MOF membrane: harvesting salinity gradient power. *ACS Appl. Mater. Interfaces* 2019, **11**: 35496–35500.

22 Li C, Wen L, Sui X, et al., Large-scale, robust mushroom-shaped nanochannel array membrane for ultrahigh osmotic energy conversion. *Sci. Adv.* 2021, **7**: eabg2183.

23 Sun Y, Wu Y, Hu Y, et al., Thermoenhanced osmotic power generator via lithium bromide and asymmetric sulfonated poly(ether ether ketone)/poly(ether sulfone) nanofluidic membrane. *NPG Asia Mater.* 2021, **13**: 1–10.

24 Yu C, Zhu X, Wang C, et al., A smart cyto-compatible asymmetric polypyrrole membrane for salinity power generation. *Nano Energy* 2018, **53**: 475–482.

25 Ji J, Kang Q, Zhou Y, et al., Osmotic power generation with positively and negatively charged 2D nanofluidic membrane pairs. *Adv. Funct. Mater.* 2016, **27**: 1603623.

26 Chen C, Liu D, He L, et al., Bio-inspired Nanocomposite Membranes for Osmotic Energy Harvesting. *Joule* 2020, **4**: 247−261.

27 Ma T, Balanzat E, Janot J M, et al., Nanopore functionalized by highly charged hydrogels for osmotic energy harvesting. *ACS Appl. Mater. Interfaces* 2019, **11**: 12578–12585.

28 Li Z, Hall A T, Wang Y, et al., Ion transport and ultra- efficient osmotic power generation in boron nitride nanotube porins. *Sci. Adv.* 2024, **10**: eado808.

29 Tunuguntla R H, Henley R Y, Yao Y C, et al., Enhanced water permeability and tunable ion selectivity in subnanometer carbon nanotube porins. *Science* 2017, **357**: 792–796.

30 Yao Y-C, Taqieddin A, Alibakhshi M A, et al., Strong electroosmotic coupling dominates ion conductance of 1.5 nm diameter carbon nanotube porins. *ACS Nano* 2019, **13**: 12851–12859.
